# Early-stage dynamics of chloride ion–pumping rhodopsin revealed by a femtosecond X-ray laser

**DOI:** 10.1073/pnas.2020486118

**Published:** 2021-03-22

**Authors:** Ji-Hye Yun, Xuanxuan Li, Jianing Yue, Jae-Hyun Park, Zeyu Jin, Chufeng Li, Hao Hu, Yingchen Shi, Suraj Pandey, Sergio Carbajo, Sébastien Boutet, Mark S. Hunter, Mengning Liang, Raymond G. Sierra, Thomas J. Lane, Liang Zhou, Uwe Weierstall, Nadia A. Zatsepin, Mio Ohki, Jeremy R. H. Tame, Sam-Yong Park, John C. H. Spence, Wenkai Zhang, Marius Schmidt, Weontae Lee, Haiguang Liu

**Affiliations:** ^a^Department of Biochemistry, College of Life Sciences and Biotechnology, Yonsei University, Seodaemun-gu, 120-749 Seoul, South Korea;; ^b^Complex Systems Division, Beijing Computational Science Research Center, Haidian, 100193 Beijing, People's Republic of China;; ^c^Department of Engineering Physics, Tsinghua University, 100086 Beijing, People's Republic of China;; ^d^Department of Physics, Beijing Normal University, Haidian, 100875 Beijing, People's Republic of China;; ^e^Department of Physics, Arizona State University, Tempe, AZ 85287;; ^f^Physics Department, University of Wisconsin, Milwaukee, Milwaukee, WI 53201;; ^g^Linac Coherent Light Source, Stanford Linear Accelerator Center National Accelerator Laboratory, Menlo Park, CA 94025;; ^h^Department of Chemistry and Physics, Australian Research Council Centre of Excellence in Advanced Molecular Imaging, La Trobe Institute for Molecular Science, La Trobe University, Melbourne, VIC 3086, Australia;; ^i^Drug Design Laboratory, Graduate School of Medical Life Science, Yokohama City University, 230-0045 Yokohama, Japan

**Keywords:** time-resolved crystallography, light-driven chloride-pumping rhodopsin, X-ray free-electron laser, serial femtosecond crystallography

## Abstract

Chloride ion–pumping rhodopsin (ClR) in some marine bacteria utilizes light energy to actively transport Cl^−^ into cells. How the ClR initiates the transport is elusive. Here, we show the dynamics of ion transport observed with time-resolved serial femtosecond (fs) crystallography using the Linac Coherent Light Source. X-ray pulses captured structural changes in ClR upon flash illumination with a 550 nm fs-pumping laser. High-resolution structures for five time points (dark to 100 ps after flashing) reveal complex and coordinated dynamics comprising retinal isomerization, water molecule rearrangement, and conformational changes of various residues. Combining data from time-resolved spectroscopy experiments and molecular dynamics simulations, this study reveals that the chloride ion close to the Schiff base undergoes a dissociation–diffusion process upon light-triggered retinal isomerization.

## Significance

Light-driven rhodopsin proteins pump ions across cell membranes. They have applications in optogenetics and can potentially be used to develop solar energy–harvesting devices. A detailed understanding of rhodopsin dynamics and functions may therefore assist research in medicine, health, and clean energy. This time-resolved crystallography study carried out with X-ray free-electron lasers reveals detailed dynamics of chloride ion–pumping rhodopsin (ClR) within 100 ps of light activation. It shows the dissociation of Cl^−^ from the Schiff base binding site upon light-triggered retinal isomerization. This Cl^−^ dissociation is followed by diffusion toward the intracellular direction. The results hint at a common ion-pumping mechanism across rhodopsin families.

Chloride ion (Cl^−^) concentration in some bacterial cells is regulated by rhodopsin proteins, generally known as halorhodopsin, or hR. These proteins use light energy to pump Cl^−^ into cells (1, 2). Light is harvested by a molecule of retinal, covalently linked to an essential lysine residue in the seventh transmembrane helix of GPCR-like (G protein–coupled receptor) proteins. Light activation causes retinal to isomerize from the all-trans to the 13-cis configuration. This change triggers subsequent conformational changes throughout the rhodopsin molecule and releases chloride into the cytoplasm. Retinal thermally relaxes to the all-trans configuration within milliseconds and is then ready for the next photocycle. Cl^−^ ions are transported from the extracellular (EC) side to the cytoplasmic (CP) side during each photocycle (3, 4).

Light-driven ion-pumping rhodopsin can be used to develop artificial solar energy harvesting and optogenetics (5–8), but the molecular mechanism must be understood in detail for such applications. Despite the importance of hR, our current experimental data concerning the structure and dynamics of the protein remain very limited. A related protein, proton (H^+^)-pumping bacteriorhodopsin (bR) discovered in the early 1970s, has been extensively studied by multiple methods, including time-resolved spectroscopy, crystallography, mutagenesis, and computer simulation (9–12). In particular, recent studies using time-resolved serial femtosecond crystallography (TR-SFX) methods performed at X-ray free-electron laser (XFEL) facilities allow three-dimensional (3D) visualization of retinal isomerization and associated local conformational changes. These changes are accompanied by movement of protons from a donor aspartate group to an acceptor aspartate (13–15). However, the central component of this process, the transported H^+^, is difficult to observe by X-ray crystallography and could not be directly traced in bR TR-SFX studies. Recently, a breakthrough was reported in a study on the sodium-pumping rhodopsin KR2 (*K. eikastus* rhodopsin 2), in which electron density signals of Na^+^ uptake were observed at Δt = 1 ms after laser illumination (16).

Cl^−^, a strong X-ray scatterer, can be directly observed from electron density maps. These maps provide first-hand information on the movement of ions as being transported within short timescales after light activation. Furthermore, hR and bR presumably share a common molecular mechanism despite transporting ions in opposite directions. A close relationship is strongly implied by the interconversion of the function of two rhodopsins. Outward H^+^-pumping bR can be converted to an inward Cl^−^ pump by changing a single residue (D85T) (17), while hR from the cyanobacterium, *Mastigocladopsis repens*, is reported to pump protons after a single mutation (T74D) (18). The chloride pump can therefore serve as a system analogous to the proton transporter and provide valuable information that is difficult to obtain directly from bR.

**Fig. 1.**
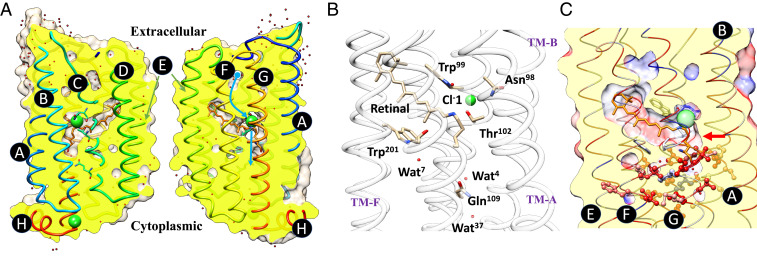
Structure of ClR and a plausible pathway of Cl^−^ transport. (*A*) Cross-sections of ClR with the backbone structure shown in cartoon representation. Transmembrane helices are marked using letters A through G, and the C-terminal helix H in the cytoplasm is also indicated. Surfaces are clipped to show the cross-section colored in yellow and the model being sliced and then opened about the axis near the helix E. Water molecules and Cl^−^ ions are shown as red- and green-colored spheres. Blue curves indicate the path of ion entering ClR and the principal pumping direction after passing retinal. (*B*) Key residues near the Cl^−^ ion and retinal, together with the NTQ motif shown in stick representation. (*C*) Residues that form a hydrophobic region between the retinal and the cytoplasm are highlighted in ball-and-stick representation. The red arrow points to a major barrier that Cl^−^ needs to overcome. ClR backbone is shown in cartoon representation, with residues colored based on hydrophobicity (the blue to red spectrum corresponds to the hydrophobicity scale from hydrophilic to hydrophobic).

In this study, we focus on chloride ion–pumping rhodopsin (ClR) from the marine flavobacterium *Nonlabens marinus* S1-08T (19). The conserved DTD motif (Asp85-Thr89-Asp96) of the bR family, residues 85, 89, and 96, is replaced by an NTQ motif (Asn98- Thr102-Gln109) in ClR (Fig. 1). The sequence identity of ClR and canonical bR from *Halobacterium salinarum* is only 27%, but the two proteins, nevertheless, have highly similar structures, including the disposition of the retinal chromophore. ClR structures at cryogenic and room temperatures clearly reveal an architecture composed of seven transmembrane helices (TM A to G) (2, 20, 21). The retinal is covalently linked to the Nζ atom of the Lys235 located on TM-G. Anomalous diffraction signals of the Br^−^ identify a stable binding site near the protonated Schiff base (PSB) and a plausible exit site on the CP side (Fig. 1*A*). Buried water molecules and locations of cavities inside ClR suggest a pathway for Cl^−^ uptake on the EC side, but the molecular mechanism for light-triggered Cl^−^ pumping remains obscure. Upon light activation, the Cl^−^ tightly held near the PSB must break free from its hydrogen bonding network (Fig. 1*B*). It then passes through a hydrophobic region to reach the CP side (Fig. 1*C*). Crystal structures of ClR were previously determined with crystals under continuous illumination of visible laser light. Intriguingly, these steady-state models revealed unexpected movement of the retinal, without indication of photo-isomerization (22). Steady-state measurements, which show averages of mixed states, are thus of limited use in deciphering the molecular mechanism of light-driven Cl^−^ pumping.

## Results

TR-SFX experiments were carried out at the Linac Coherent Light source (LCLS) at SLAC National Accelerator Laboratory in Menlo Park, CA. Microcrystals of ClR proteins were excited by 100-femtosecond (fs) 550 nm laser pulses (pulse energy = 7.91 μJ, corresponding to a peak fluence of 0.90 mJ/mm^2^). Crystals were then probed with X-ray pulses a short time after excitation. A total of five atomic resolution structures of ClR were determined from the TR-SFX data for the dark state and at four time delays (Δt) of ∼1, 2, 50, and 100 ps (picosecond, 10^−12^ s). These structures showed that retinal isomerization occurs in 1 ps and gradually relaxes to a stable 13-cis configuration within 100 ps. Residues and water molecules exhibit coordinated movements to accommodate isomerization. Most importantly, the Cl^−^ ion near the PSB first moves toward the EC space upon retinal isomerization and then moves toward Thr102 of the TM-C, revealing a dissociation–diffusion process. These results visualize 3D conformation changes in ClR after photoactivation. This finding is a critical step for a complete understanding of the Cl^−^ transport process, which may help unify the light-driven ion-pumping mechanism of rhodopsin families.

### Relaxation of All-Trans to 13-Cis Retinal Isomerization.

**Fig. 2.**
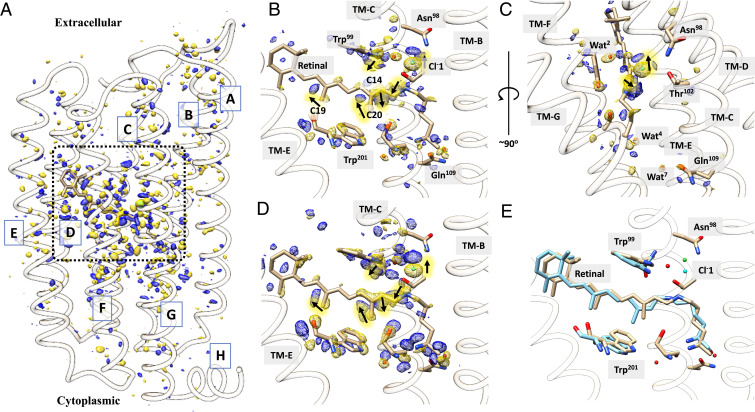
Refined models and Fourier DMAPs. (*A*) Observed DMAP for Δt = 1 ps at a 3.5 σ contour level. (*B*) Observed DMAP in the vicinity of retinal (contour level as in *A*). (*C*) View along the retinal (contour level as in *A*). Positive and negative electron density pairs are indicated with black arrows. (*D*) DMAP calculated from refined light and dark structures (contour level of 7.0 σ). (*E*) Structure (cyan) at Δt = 1 ps superimposed on the structure of the dark state (tan).

**Fig. 3.**
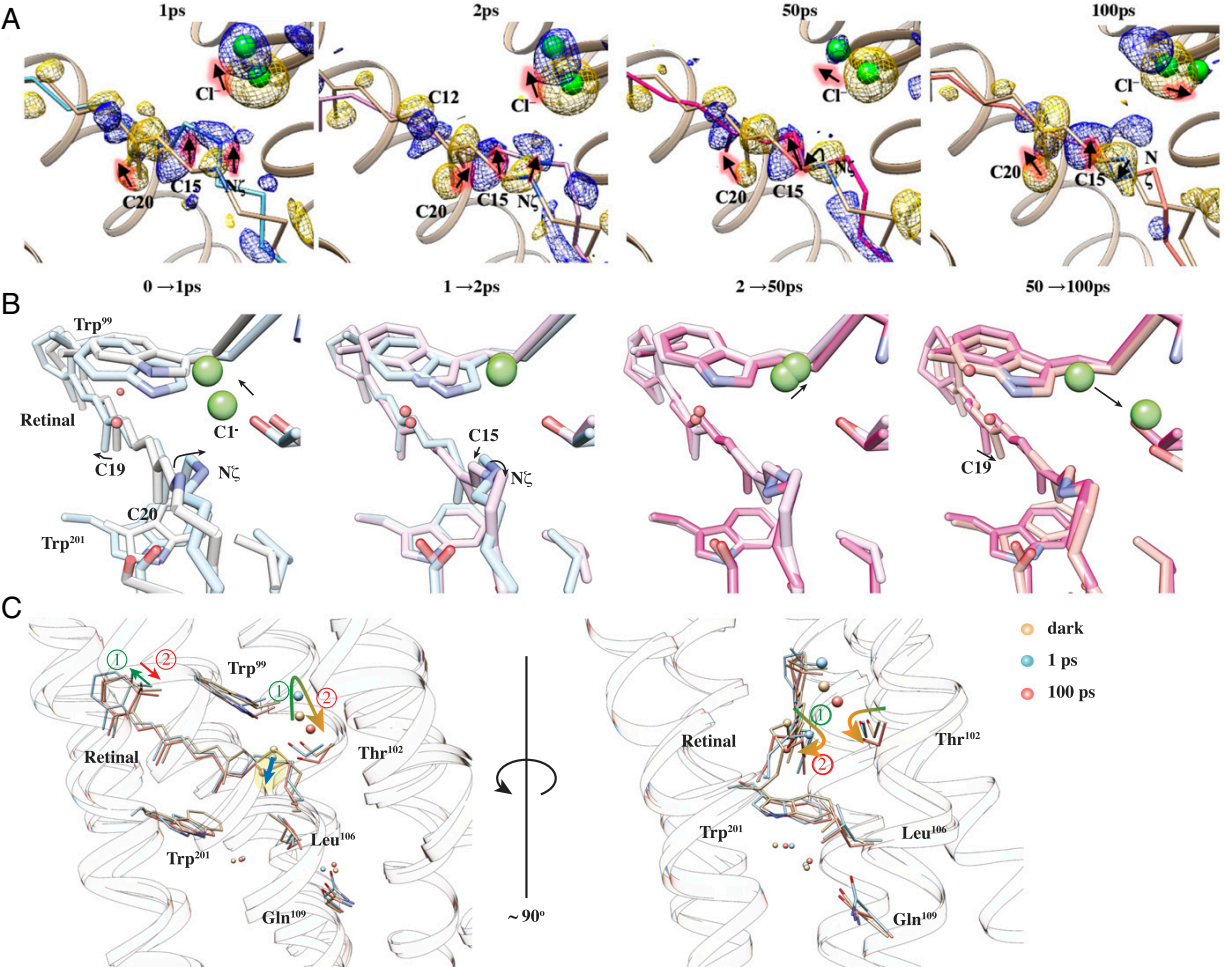
Fourier DMAPs near the retinal and associated conformational changes. (*A*) Retinal bound to Lys235 overlaid with DMAPs at time delays from 1 to 100 ps. DMAPs are contoured at 4.0 σ; positive and negative densities are shown in blue and gold colors, respectively. The structure of the dark state (tan color) is shown as a reference; conformations at 1, 2, 50, and 100 ps are labeled and shown in different colors. (*B*) Conformational changes near the retinal from a perspective that depicts the Schiff base, nearby residues, and the Cl^−^. Structures at consecutive time points are compared to illustrate the dynamical process. (*C*) Models of dark and two time points after activation (1 and 100 ps) are shown to summarize the movements of the retinal and Cl^−^1. The arrows indicate major displacements. Circled numbers mark the two major stages of conformational changes.

Fourier difference density maps (DMAPs) between photoactivated and dark states were calculated for the four time delays using the TR-SFX data (see *Materials and Methods*). Strong difference density features are localized in the vicinity of the retinal, indicating that, on picosecond time scales, larger coordinated conformational changes have not yet migrated throughout the ClR molecule (see Fig. 2*A* for the overall view of DMAP at Δt = 1 ps; DMAPs for other datasets are included in *SI Appendix*, *Supplementary Materials*). DMAPs in the vicinity of the retinal are shown in Fig. 3 for each time delay. The strongest negative signals are associated with the movement of Cl^−^ near the PSB (up to −15.0 σ). This Cl^−^ ion is termed as Cl^−^1 to be distinguished from the Cl^−^ ion near the exit site. Paired positive densities around the negative peak indicate that Cl^−^1 becomes more mobile and moves away from the PSB toward the EC side. Strong peaks of paired negative and positive densities near C14, C15 of the retinal, and the Nζ atom of the Lys235 are explained by the transformation from an all-trans retinal to its 13-cis isomer (Fig. 2 *B*–*D*). The protein and chromophore were refined as a single conformation against extrapolated structure factors for each time delay (see *Materials and Methods*). The dark-state structure was refined to 1.65 Å, and the four photoactivated state structures were refined to 1.85 Å (*SI Appendix*, Table S1). The 13-cis retinal becomes slightly elongated and twisted immediately after light activation (Fig. 2*E*).

Retinal isomerization is evident at 1 ps after illumination, as reflected on the torsion angle of C12-C13 = C14-C15. According to the refined structures, this angle flips from about 179° in the dark state to 4° at 1 ps. Atom C15 moves toward the EC face of the protein, accompanied by a sideways shift toward TM-C (Fig. 3). The torsion angle of C14-C15 = Nζ-Cε flips as a result from 155° (dark) to −65° (1 ps), and the retinal continues to change, showing a torsion angle of 187° at 2 ps and 126° at 100 ps. Furthermore, at 100 ps, the C12-C13 = C14-C15 torsion angle relaxes to about −7° and the β-ionone ring has almost returned to its dark-state position (Fig. 3*C*). At 1 ps, the retinal and associated lysine sidechain deviates from the dark state by 1.1 Å rmsd, mainly due to isomerization that twists and elongates the chromophore. The distance between the Nζ atom and the centroid of the ionone ring increases from 12.7 Å (dark state) to 13.1 Å at 2 ps and then shrinks to about 12.2 Å as the retinal relaxes into the 13-cis configuration at 100 ps. The chromophore rmsd is 1.2 Å at 50 ps and 0.9 Å at 100 ps, relative to the dark-state structure. While these model-derived parameters also reflect the finite resolution of the crystal structures and diffusive nature of energy spread from the photoexcited retinal, the overall conformational changes around the chromophore are quantitatively captured via TR-SFX. Cl^−^1 dissociates from the PSB and moves toward the EC direction at 1 ps, and then it moves toward Thr102 (Fig. 3*C*). The Nζ movement is essentially a rotation around the retinal long axis to complete isomerization.

### Movements of Cl^−^ and Its Interactions with Residues Near PSB.

**Fig. 4.**
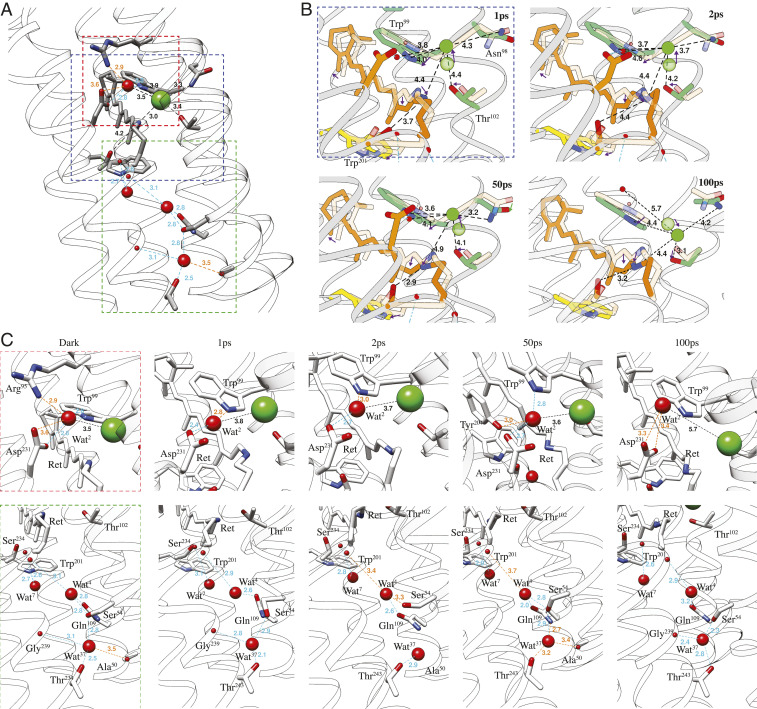
Detailed dynamics of Cl^−^ and key water molecules in ClR. (*A*) Binding of Cl^−^ near the PSB and water-mediated hydrogen bonds in the dark state. (*B*) Dynamics of Cl^−^ and distances (in Å) from nearby residues. Arrows indicate directions of movement. (*C*) Progression of water-mediated hydrogen bonds from the dark state to 100 ps after flash illumination. Blue and orange colors indicate stable hydrogen bonds and relaxed hydrogen bonds with numbers marking distances in Å.

Key interactions of Cl^−^1 with the protein at each time point are shown in Fig. 4, and distances are listed in *SI Appendix*, Table S2 for all structures. In the dark state, Cl^−^1 forms hydrogen bonds with the PSB and Thr102, but both bonds are broken at 1 ps as the retinal isomerizes. Thr102 remains nearly static, while the distance between OG1 of Thr102 and the Cl^−^ increases from 3.4 Å to 4.4 Å. Cl^−^1 moves 1.3 Å away from the retinal and toward the EC face of the protein, a movement tracked by the sidechain of Asn98 (Fig. 4*B*). The distance between Cl^−^1 and ND2 of Asn98 increases from 3.3 Å to 4.3 Å at 1 ps and then reduces to 3.7 Å at 2 ps as the sidechain rotates. Wat2 is located about 3.5 Å from Cl^−^1 in the dark state and jumps at 1 ps in broadly the opposite direction to the Schiff base. Concomitantly, the indole ring of Trp99 rotates by 14.9° and maintains a distance of 3.6 to 3.8 Å between Nε1 and Wat2. The interaction network around Cl^−^1 is therefore strongly disrupted within 1 ps by movements of both Cl^−^1 and the PSB. Notably, Leu106 moves about 1.5 Å away from Trp201, creating a wider gap between TM-C and TM-F (Fig. 3*C*).

DMAP at 50 ps shows a considerable positive peak on the CP side of dark-state Cl^−^1 at the 3 σ level; however, the refined position of Cl^−^1 at 50 ps is similar to the positions at 1 and 2 ps. Trp99 has largely rotated back to its dark-state position by 50 ps, but the retinal exhibits little change from its 2 ps structure. At 100 ps, Thr102 has shifted toward the cytoplasm by 0.5 Å, making space for Cl^−^1 to move in this direction. Accordingly, the position of Cl^−^1 was refined toward the newly emerged positive peak at 100 ps. The presence of multiple positive peaks centered around the negative peak of dark-state Cl^−^1 indicates that the Cl^−^ becomes highly mobile after retinal isomerization. Motions of Cl^−^1 and retinal as well as nearby residues have been compiled into a 3D molecular movie (*SI Appendix*). The movie shows that Cl^−^ motion follows a dissociation–diffusion process in the first 100 ps after illumination. Specifically, the Cl^−^ ion first dissociates from the PSB upon retinal isomerization and remains attracted to Trp99 and Asn98. It then diffuses toward Thr102, marking the start of ion transit through the hydrophobic region.

### Dynamics and Roles of Water Molecules.

Wat2 lies next to the PSB at the center of a hydrogen bonding network formed by Arg95, Tyr204, and Asp231. Arg95 breaks its bonds with both Wat2 and Asp231 at 1 ps, releasing the water molecule (Fig. 4*C*). The distance between Cl^−^1 and Wat2 is about 3.8 Å as Cl^−^1 moves away from the PSB. Paired positive density in the difference density map is weaker than negative density, suggesting that Wat2 becomes less ordered. The hydrogen bond network with nearby residues is shown in Fig. 4*C* for all structures. As Cl^−^1 moves toward Thr102 at 100 ps, Wat2 completely separates from the ion. Wat2 at 100 ps mainly interacts with Asp231 and Trp99 Nε1 and is less ordered than in the dark state.

Only three internal water molecules are found between the chromophore and the cytoplasm in contrast to the much more hydrated internal cavities toward the EC face of ClR. These three water molecules lie between TM-B, C, and G (lower panel of Fig. 4*C*). In the dark state, Wat4 forms hydrogen bonds with the carboxyl oxygen of Lys235 (TM-G) and the sidechains of Ser54 (in TM-B) and Gln109 (in TM-C) of the NTQ motif. Wat4 is close to the center of gravity of the molecule. This water does become mobile, although it does not move significantly within the first 100 ps after illumination. The hydrogen bond with Ser54 is quickly broken and reforms at about 50 ps. Wat4 may therefore help transfer the excitation from the chromophore to other parts of ClR. Wat7 forms hydrogen bonds with the carboxyl of Ser234 and the sidechain of Trp201, which lies against the retinal. The retinal presses against Trp201 upon light activation and increases the distance between Wat7 and Trp201 from 2.7 Å to about 3.4 Å. Located about 5.0 Å further toward the CP face of the protein than Wat4, Wat37 forms hydrogen bonds with sidechains of Ser54 and Thr243 as well as the carboxyl of Gly239. Wat37 moves toward the carboxyl upon illumination and jumps back to its resting position by 50 ps.

### Transmembrane Helix Deformation.

**Fig. 5.**
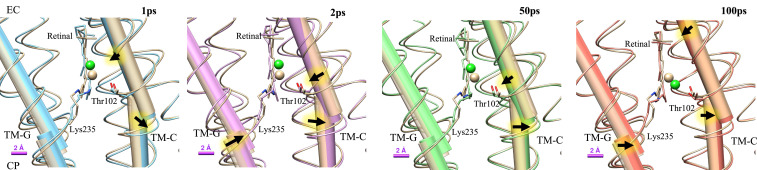
Conformational changes of TM-C and TM-G helices. The TM-C and TM-G helices kink near Thr102 and Lys235, respectively. Dark-state structures of TM-C and TM-G are shown in tan color. At 1 ps, the kink is more pronounced for both TM-C and TM-G. At 100 ps, TM-G moves back to its dark state conformation, while the EC segment of the TM-C bends inwards and the CP segment tilts outwards. Arrows indicate directions of motion relative to the dark state. The Cl^−^, retinal, and Thr102 are shown to illustrate the relation between retinal isomerization and helical deformation.

The refined structures indicate that major conformational changes are localized near the retinal, consistent with the strongest features in the DMAPs. Isomerization results in both a downward shift of the retinal and Lys235 toward the CP side and a lateral translocation toward TM-C. The Cα atom of Lys235 moves by about 0.6 Å at 100 ps toward its Cβ atom, suggesting a force exerted by the retinal. TM-G kinks slightly at Lys235 on activation, with the angle between the two halves of TM-G increasing from about 4.6° in dark state to 4.8° at 1 ps and to 5.4° at 2 ps. The kink angle relaxes to 5.2° at 100 ps, but TM-G maintains its translocation toward TM-C (Fig. 5). The EC and CP helical segments of TM-C, separated by Thr102, move in opposite lateral directions. The EC half of TM-C moves closer to the central axis of ClR and shifts toward the CP side; the CT half tilts outward near Thr102. Movements of TM-C indicate that the ion channel closes on the EC side and that the cavity near the PSB expands. Leu106 on TM-C shifts away from the TM-G after light activation. This displacement may be related to the opening of the hydrophobic gate. Leu106 is a major hydrophobic residue on the pathway to the cavity near the conserved residue Gln109. The channel between Cl^−^1 and the cavity near Gln109 is blocked in the dark state. The sidechain of Leu106 moves away from TM-G by about 0.6 Å at 100 ps. If this passage is further enlarged in later stages of the ClR photocycle, Cl^−^ can migrate to the region near Gln109.

## Discussion

Although the resolution of the data prevents us from safely fitting more than one molecular conformation for each dataset, mixtures of intermediates may be present at each time point after illumination (*SI Appendix*, Fig. S10). While angles and distances could be derived from the fitted atomic models, caution requires that any structural conclusions drawn from the data must also agree with features in the DMAPs. In particular, the Cl^–^ ion occupies multiple sites after activation according to the data. At the 1 to 2 ps time points, the location of Cl^–^ is close to Trp99, reflecting the dissociation from the PSB after photoactivation. At 50 to 100 ps, stronger Cl^–^ signals appear close to Thr102. Although the inclusion of an alternative position for Cl^–^ can improve the agreement the density peaks around Cl^–^, the structure of the retinal and the ClR protein is essentially identical to the case when only a single Cl^–^ position is considered. Despite the limitations of the structural refinement, TR-SFX data in this study allow several important conclusions to be drawn, such as rapid activation of the chromophore and the spread of the signal over tens of ps. The motion of Cl^–^ is also evident from the experimental data. Featureless difference maps (*SI Appendix*, Fig. S10*B*) between experimental DMAP and the computed DMAP from atomic models show that each atomic model represents the dominant structure in an ensemble of evolving intermediate states. Mixtures can be identified and separated once a time series of more finely spaced time delays becomes available (23–25).

### Physical Interpretation of ClR Dynamics.

The dipole switching model (26) predicts that Cl^−^ will follow the motion of the PSB and move to the opposite side of the retinal as it isomerizes, but the results presented here are different. This TR-SFX study shows that isomerization is too rapid for Cl^−^ to react on the same time scale and it becomes dissociated from the PSB. Ultrafast isomerization abruptly reduces electrostatic interactions between the PSB and Cl^−^, while forces from other residues remain unchanged, resulting in a net force pulling Cl^−^ toward the EC side (“upwards” in *SI Appendix*, Fig. S12). The new configuration does not form a well-coordinated binding site for Cl^−^, so it diffuses toward Thr102, as observed between 50 and 100 ps. Upward motions of Cl^−^ in ClR and of Wat402 in bR reported by Nogly et al. (14) are consistent and reflect the sudden loss of the bonding with the PSB. The enhanced mobility of the Cl^−^ in ClR with a 13-cis retinal is validated using molecular dynamics simulations. The Cl^−^ ion hardly moves in ClR with an all-trans retinal and maintains a close distance (3.0 to 3.5 Å) to the PSB over 100 ps. In contrast, starting with the same ClR structure except with the retinal in its 13-cis configuration, the Cl^−^ ion quickly moves away from the PSB, showing a broader distribution of Cl^−^-Nζ distance observed in simulations (*SI Appendix*, Fig. S11). The Cl^−^ ion did not enter the hydrophobic region (depicted in Fig. 1*C*) even when extending simulations to 1.0 ns. To reproduce the retinal-triggered conformational changes, it may require simulating the process of isomerization using advanced protocols, such as quantum mechanics (QM) or QM/MM (molecular mechanics) hybrid simulations.

Combining kinetic information obtained from time-resolved spectroscopy experiments with conformational changes revealed by TR-SFX is crucial to fully understand the molecular mechanism. The complex kinetics of ClR up to 100 ps from photoactivation were probed using time-resolved absorption spectroscopy, showing that activated ClR relaxes to the K-state of the photocycle within this time frame (*SI Appendix*, Fig. S2 for the kinetics model). The results also indicate that the structures at 1 to 2 ps time delays are mixtures of electronic excited state and the hot 13-cis state, while the structures at 50 to 100 ps correspond to the relaxed 13-cis state. However, finer sampling of time domain using TR-SFX is required to separate the mixed states, even if they possess distinct conformations.

### Pumping Laser Wavelength and Power.

The ClR absorption spectrum of the dark state features a broad peak centered around 550 nm (*SI Appendix*, Fig. S2*A*). Time-resolved absorption spectroscopy experiments were carried out with light of either 480, 540, or 550 nm wavelength, but the spectra and subsequent analysis were consistent in each case. To reduce the excess heat exerted on retinal and ClR, a 550 nm femtosecond laser was used in TR-SFX experiments. For the spectroscopy experiments, a 480 nm pump laser was used to avoid the overlapping with the isosbestic point in transient spectra (*SI Appendix*, Fig. S2*D*). The power dependency was measured using transient absorption spectroscopy to identify the linearly responding regime (*SI Appendix*, Fig. S2*B*). For the time delay of 100 ps after flash illumination, four power levels of the pumping laser were applied to cross-validate the signals. The features in the Fourier DMAPs indicate that major conformational changes at 100 ps are consistent for the four power levels of the pumping laser, providing additional justification on the choice of the pumping laser power (*SI Appendix*, Fig. S9).

### Comparison to Other Rhodopsins and Indication of a Unified Pumping Mechanism.

**Fig. 6.**
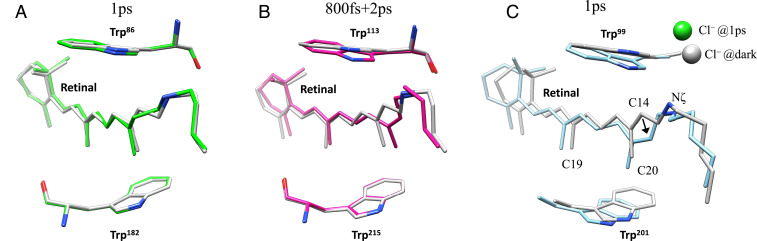
Conformational change comparisons of the retinal and two nearby tryptophan residues in three rhodopsins. (*A*) bR (PDB 6GA4) (15); (*B*) KR2 (PDB 6TK7 for dark and 6TK5 for 800 fs + 2 ps) (16); and (*C*) ClR. In each panel, the corresponding dark-state structure is shown in gray as a reference.

Retinal isomerization in ClR is similar to that in KR2 (16), with the C20 methyl group tilting toward TM-C instead of TM-G as in bR (Fig. 6). The more pronounced retinal movements in ClR, compared with bR or KR2, indicate that larger conformational changes might be essential for pumping Cl^−^, whose radius is larger than the radius of Na^+^ (1.81 Å versus 1.02 Å in terms of effective radii). The unique packing configuration of retinal in ClR can explain the ultrafast conformational changes observed. The positive charge of the PSB in KR2 and bR is countered by nearby aspartate side chains (Asp116 and Asp85, respectively), but in ClR, the counterion is the substrate chloride itself. Each of these negative charges helps to stabilize all-trans retinal in the dark state, and retinal isomerization increases the distances between PSB and its counter ions. The size and speed of the conformational changes on photoactivation are also consistent with the functional behaviors of the three rhodopsins. Na^+^ does not enter KR2 until milliseconds after the initiation of a photocycle, so there is no need for large conformational changes on a picosecond time scale. Protons are extremely small and easily cross hydrogen-bond networks, so large conformational changes are not required for bR either. However, the hydrophobic barrier between the PSB and CP face of ClR may require further pore opening that involves rearrangements of transmembrane helices. The kinks on TM-C and TM-G observed in this study may be part of such conformational changes; however, detailed dynamics can only be revealed by time-resolved crystallography experiments designed for longer time delays. Furthermore, the reverse directions of ion transport and the opposite charges suggest a phase shift in the photocycle of ClR comparing to that of bR or KR2. The Cl^−^ release and the uptake of H^+^/Na^+^ may correspond to similar structural conformations, reflected by the opening of the CP face of the protein. It is possible that early-stage dynamics of ClR will throw light on the later stages of the photocycle in bR or KR2. By complementing these highly studied rhodopsin systems, ClR may help develop a unified molecular model of light-driven ion pumping by rhodopsins.

## Materials and Methods

### Chloride Ion–Pumping Rhodopsin Crystallization.

The ClR gene (GI: 594833795) from *Nocardioides marinus* in the pET21b vector was transformed into *Escherichia coli* BL21-CodonPlus (DE3; Agilent Technologies), and the cells were grown in high-salt Luria–Bertani medium at 37 °C. When the optical density at 600 nm (OD_600_) was over 1.0, 50 μM all-trans retinal (Sigma Aldrich) and 0.5 mM isopropyl-β-d-thiogalactoside were added to induce ClR expression for 6 to 8 h at 30 °C. Harvested cells were lysed by sonication in lysis buffer containing 50 mM Tris HCl (pH 7.0) and 150 mM NaCl. The membrane fraction was isolated by ultracentrifugation (Beckman) at 370,000 × *g* for 40 min at 4 °C; resuspended in solubilization buffer containing 50 mM Tris HCl (pH 7.0), 150 mM NaCl, 1% n-dodecyl-β-d-maltoside (DDM), and 0.2% cholesteryl hemisuccinate (CHS); and incubated for 2 h at 4 °C for solubilization. The solubilized protein was purified by TALON affinity chromatography, and the eluate was applied to a Superdex-200 size-exclusion column (GE Healthcare) equilibrated with buffer containing 20 mM Hepes (pH 7.5), 150 mM NaCl, 0.05% DDM, and 0.01% CHS.

The purified protein was mixed with monoolein (1-oleoyl-rac-glycerol; 9.9 MAG) at a 1:1.5 molar ratio (volume/volume) using a syringe lipid mixer (Hamilton). After formation of a clear lipidic cubic phase (LCP), ∼5 µL the protein-laden LCP sample was injected using a 100 µL syringe filled with 55 to 60 µL precipitant solution containing 0.15 M sodium chloride, 0.15 M calcium chloride, 0.1 M MES (pH 6.0), and 30% polyethylene glycol dimethyl ether 500. Microcrystals of ClR were grown in the syringes, and red-colored crystals were identified using a microscope (*SI Appendix*, Fig. S1*A*). Prior to LCP–XFEL data collection, ClR crystals grown in syringes were incubated in dark conditions at 25 °C for 1 h. Approximately 40 µL LCP sample was transferred into a new syringe after removing the precipitant solution, and ∼10 µL 9.9 MAG was applied to the LCP sample and homogenized. The sample was transferred into an LCP injector through an LCP loading needle.

### Pump–Probe Experiments at LCLS.

The microcrystals of ClR were freshly prepared 2 d prior to the experiments. They were delivered to the XFEL beam path in the main chamber at the Coherent X-ray Imaging (CXI) beamline (27, 28) using an LCP injector (29). Before exposure to XFEL pulses, the crystals were illuminated by femtosecond (pump) laser pulses at a wavelength of 550 nm. Crystals were then probed using 9.54 keV (λ = 1.30 Å) XFEL pulses, focused to ∼1 µm full width at half maximum (FWHM). The Cornell-SLAC Pixel Array Detector (30) was placed ∼90 mm from the interception point, allowing diffraction up to 1.64 Å resolution to be recorded at the edge of the detector. Full SFX datasets were collected for four time delays that covered a time range from 1 to 100 ps after laser excitation. An interleaved “light–dark” data collection mode was used, as the pumping laser illuminated the flowing samples at 60 Hz and the X-ray laser was operated at 120 Hz (*SI Appendix*, Fig. S1*B*). With this mode, the pump laser pulses are about 16.7 ms apart; this setup reduces light contamination from a previous laser pump pulse. Furthermore, the microcrystals of ClR were injected to the experimental chamber at a high flow rate such that microcrystals traveled a distance of ∼200 µm between pump laser pulses. With this spacing, the energy density of the previous pumping pulse was reduced to about 0.6% of its peak value. We used the chosen flow rate to optimize the data quality and the experimental throughput.

A set of diffraction data for the dark state were collected separately (with pump laser off) to obtain a high-quality reference dataset. The pumping laser has pulse durations of about 100 fs, with a beam size of about 150 μm FWHM centered at the sample position. The XFEL pulse duration was about 50 fs, estimated from the electron pulse duration.

Absorption spectroscopy was used to optimize both the wavelength and the power of the pump laser and to assess nonlinear effects that might interfere with the outcomes of the TR-SFX experiment (*SI Appendix*, Fig. S2, and see the following section). By varying the pump laser power, we found that the linear regime of the transient absorption signals extended to about 0.48 mJ/mm^2^ after calibrating to the pumping laser durations used for TR-SFX (FWHM of ∼100 fs in TR-SFX versus ∼35 fs in spectroscopy experiments). Considering that the LCP medium may scatter a portion of light and that the samples are in a crystalline state, we chose to use a pumping laser at 0.90 mJ/mm^2^ for TR-SFX. For the time delay of 100 ps, three additional pumping laser power levels (0.17, 2.63, and 6.49 mJ/mm^2^) were applied to cross-validate the activation effects.

### SFX Data Analysis.

The overall workflow for data analysis is summarized in *SI Appendix*, Fig. S3. Raw data were screened using Cheetah (31) and ClickX (32) to identify the patterns with sufficient diffraction signals. The timing tool (33) was applied to bin the dataset of 1 ps nominal delay into two windows, averaged at 1 and 2 ps time delays (*SI Appendix*, Fig. S1*C*). After the first round of screening, about 10% of the raw data were identified as diffraction patterns. Subsequently, diffraction patterns were indexed, scaled, and merged with the CrystFEL software suite (version 0.8.0) (34). Multiple indexing algorithms were used to improve data quality. The indexing rate varied from 60 to 90%, and as a result, between 22,000 and 47,000 diffraction patterns were indexed for each dataset. This yielded complete datasets with high redundancy and signal-to-noise ratios (*SI Appendix*, Table S1). The partialator program in the CrystFEL was used to merge diffraction signals into final intensity data with unity partiality model.

The dark-state structure was determined at a resolution of 1.65 Å from 37,044 indexed patterns, providing a high-resolution reference model. Fourier DMAPs between pumped and dark states were calculated using methods reported previously (35, 36) that employ a weighting scheme to suppress structure factor outliers. Because only a fraction of ClR proteins could be pumped into the photocycle, the structures at each time point were determined with extrapolated structure factors based on a method successfully used with data from previous TR-SFX experiments (37–39).

The DMAP for each time delay was calculated from the structure factor differences between the pump and dark datasets (i.e., Δ|F| = |F_pump_| − |F_dark_|). The dark model phase information, Φ_dark_, was used to calculate the Fourier DMAPs: Δ$\rho \left(r\right)=FT\left\{\Delta \left|F\right|{\text{e}}^{-\text{i}(2\pi hr+{\text{Φ}}_{dark}\left(h\right))}\right\}$ (*SI Appendix*, Figs. S4, S6, and S7).

To refine structures from signals of DMAPs, extrapolated electron density (ED_ext_) maps were used. For extrapolated structure factor (*F*_ext_), the amplitudes of a multiple of the |ΔF| are added to the |*FC*_*dark*_|, such that the $|F_{ext}|=\left|FC_{dark}\right|+N_{ext}*\Delta \left|F\right|$, which is combined with the reference state (dark) phases Φ_dark_. Here, the use of |*FC*_dark_| derived from an accurately refined dark-state model are preferred over the |F_dark_|, as explained by Terwilliger and Berendsen (40). From the phased ***F***_*ext*_, ED_ext_ maps were calculated with the CCP4 program “fft” (41). A characteristic, N_ext_, is established when the electron density in the ED_ext_ at the positions with strong negative features in DMAPs just vanishes. When N_ext_ is too large, false-negative features will appear in the ED_ext_. This can be visualized by summing up negative values in the ED_ext_ within a volume that contains strong features in the DMAPs. In ClR, the electron densities around the dark-state positions of C13, C14 of the retinal, and Cl^−^1 are used to determine the N_ext_. *SI Appendix*, Fig. S5 shows the results for such a summation for all time delays, with N_ext_ found to be around 22 for the main datasets with a pumping laser pulse power of 0.90 mJ/mm^2^. For the control datasets, the extrapolation factors are 18 and 28 for the cases with 0.17mJ/mm^2^ and 6.49 mJ/mm^2^ pumping laser energies, respectively. The extrapolation factor N_ext_ is approximately related to the population transfer (PT): $PT∼\frac{100}{N_{ext}}*2$ [%], where the factor of 2 accounts for the difference Fourier approximation. For determined N_ext_, structural models were determined from the resulting ED_ext_ maps. The dark model was used as an initial model for a refinement against the ED_ext_ map by a stepped real-space refinement in Coot (42) with the torsional restraint switched off (default in Coot). In order to facilitate structural interpretation and refinement, a new residue, RLY (Retinal-LYsine), was introduced to represent the retinal cross-linked with lysine. Restraints for RLY were prepared using the eLBOW program in PHENIX (43, 44). Positions of Cl^–^ and water molecules were also refined using the real-space refinement in Coot.

After real-space refinement, a new structural model for this time delay is obtained. From the dark-state model and the real-space–refined pumped model, calculated Δ***F***_*calc*_ is determined with both amplitude |Δ***F***_*calc*_| and phases ϕ_Δ_. The prominent features match well between the DMAP_calc_ calculated from the phased difference structure factors compared to the DMAP_obs_ (*SI Appendix*, Fig. S4). The ϕ_Δ_ were combined with the measured Δ|F_obs_| to obtain the phased extrapolated structure factors, PF_ext_, for the model at time delay Δt, where PF_ext_ = FC_dark_ + N_ext_ x ΔF_obs_, and the summation is done in the complex plane with ΔF_obs_= Δ|F_obs_| e^-iϕΔ^. The structure at Δt was then refined against the |PF_ext_| using restrained reciprocal space refinement with phenix.refine (44) (*SI Appendix*, Fig. S8). Figures were generated with Chimera (45) and Pymol (46).

The derived quantities, such as distance, angles, and torsion angles, are calculated using Chimera programs. In particular, hydrogen bonds were determined based on the geometry of atomic structures. The distance/angle between donor and acceptor were used to identify hydrogen bonds. The default criteria used in UCSF Chimera program based on a survey of high-resolution structures was adapted in the hydrogen bonding analysis (47). The hydrogen bonds that meet strict criteria are recognized as stable hydrogen bonds, while those with looser tolerances are defined as relaxed hydrogen bonds.

### Molecular Dynamics Simulations.

The ClR system was equilibrated in 1-palmitoyl–2-oleoyl-sn-glycero-3-phosphocholine solvated in a water box. The PPM server (48) was used to align the transmembrane portion to the *z*-axis. CHARMM-GUI (49) was used to set up simulation systems, with one ClR molecule solvated in a water box containing 150 mM NaCl. The system was modeled using the CHARMM36 force field (50). The constant pressure and temperature (NPT) ensemble (at 1 atm pressure and room temperature of 293.15 K) was simulated using GROMACS 5.1.2 (51). Lysine covalently bound to retinal was represented using engineered residue-type RLY generated with the CHARMM-GUI. Conjugate gradient algorithm was utilized to minimize the models prior to the production MD (molecular dynamics) simulation runs. The energy-minimized systems were equilibrated for 0.25 ns with positional restraints on nonhydrogen atoms of the ClR system (protein, retinal, and chloride ions) under 1 atm pressure. The harmonic restraining force constant was gradually reduced from 400 kJ/(mol · nm^2^) to 40 kJ/(mol · nm^2^) during the 0.25 ns equilibrium simulations. Then, 10 trajectories (each 1.0 ns, starting from the equilibrated systems with different random velocities that follow the Maxwell–Boltzmann distribution) were simulated for each system with either an all-trans or a 13-cis retinal. The integration step size was set to 0.002 ps. The VMD program (52) was used to visualize the simulation trajectories, and the distances were extracted using the GROMACS program. The information from the first 100 ps of each trajectory is used to calculate the atomic distances. The extended simulations were analyzed to predict longer timescale dynamics.

### Time-Resolved Absorption Spectroscopy.

Ultraviolet–visible (UV–Vis) absorption spectra were collected on an Agilent Technologies Cary 60 UV–Vis spectrophotometer at room temperature using a 1 mm thick quartz cuvette. ClR protein solution (60 mg/mL ClR protein sample was mixed with monoolein at a 1:1.5 molar ratio [volume/volume]) was excited with femtosecond pulses and then probed with a time-delayed supercontinuum. We set the relative polarization of the pump and probe to the magic angle to eliminate rotational dynamics from signals. Pump pulses were generated by pumping an optical parametric amplifier with 2.4 to 2.6 mJ pulses extracted from a Coherent Astrella regenerative amplifier (800 nm, 35 fs FWHM, 1 kHz, and 7 W). The supercontinuum was generated using a small amount of 800 nm light from the regenerative amplifier, further attenuating with a half-wave plate and polarizer combination and then focusing into a sapphire window. To investigate the pumping laser wavelength dependency, the experiment was repeated with pumping laser wavelengths of 480, 540, and 550 nm. The power titration was carried out for pulse energies up to 0.24 mJ/mm^2^, and the transient absorption spectroscopy (TAS) signals (milli Optical Density, mOD) at 1.5 ps time delay in the wavelength of 580 to 620 nm were computed to assess the linear responding region (*SI Appendix*, Fig. S2*B*).

To reduce the pumping laser influence to the TAS signals, the data with 480 nm pumping laser (∼3 mW power) was used to investigate the kinetics. Transient absorption data were collected with time delays between pump and probe pulses up to 1,000 ps in the spectrum range of 500 to 850 nm. The TAS data are interpreted with a five-state kinetic model (15, 53, 54) using the global and target fitting analysis method (55). Time-resolved TAS data were measured for ClR crystals. The crystals were manually located and focused to the optical path for TAS measurements. Due to the technical difficulties and crystalline sample stability, high-quality TAS data were measured with ClR crystals only at three time delays (1, 6, and 11 ps). The comparison of TAS data of ClR in solution and in crystals indicates that the kinetics of ClR are not altered by crystal packing (*SI Appendix*, Fig. S2*G*).

## Supplementary Material

Supplementary File

Supplementary File

Supplementary File

Supplementary File

Supplementary File

Supplementary File

Supplementary File

Supplementary File

Supplementary File

Supplementary File

## Data Availability

The structures and the associated structure factors are deposited in the Protein Data Bank (PDB) under the following access codes: 7CRJ (dark state), 7CRI (1 ps), 7CRK (2 ps), 7CRL (50 ps), 7CRS (100 ps, 0.90 mJ/mm^2^), 7CRT (100 ps, 0.17 mJ/mm^2^), 7CRX (100 ps, 2.63 mJ/mm^2^), and 7CRY (100 ps, 6.49 mJ/mm^2^). The structures and density maps are available at the GitHub repository: https://github.com/LiuLab-CSRC/Clr-Dynamics (56).

## References

[1] K. Inoue, F. H. M. Koua, Y. Kato, R. Abe-Yoshizumi, H. Kandori, Spectroscopic study of a light-driven chloride ion pump from marine bacteria. J. Phys. Chem. B 118, 11190–11199 (2014). 25166488 10.1021/jp507219q

[2] T. Hosaka., Structural mechanism for light-driven transport by a new type of chloride ion pump, *Nonlabens marinus* rhodopsin-3. J. Biol. Chem. 291, 17488–17495 (2016). 27365396 10.1074/jbc.M116.728220 PMC5016146

[3] L. O. Essen, Halorhodopsin: Light-driven ion pumping made simple? Curr. Opin. Struct. Biol. 12, 516–522 (2002). 12163076 10.1016/s0959-440x(02)00356-1

[4] O. P. Ernst., Microbial and animal rhodopsins: Structures, functions, and molecular mechanisms. Chem. Rev. 114, 126–163 (2014). 24364740 10.1021/cr4003769 PMC3979449

[5] A. K. Sharma, J. L. Spudich, W. F. Doolittle, Microbial rhodopsins: Functional versatility and genetic mobility. Trends Microbiol. 14, 463–469 (2006). 17008099 10.1016/j.tim.2006.09.006

[6] V. Gradinaru, K. R. Thompson, K. Deisseroth, eNpHR: A Natronomonas halorhodopsin enhanced for optogenetic applications. Brain Cell Biol. 36, 129–139 (2008). 18677566 10.1007/s11068-008-9027-6 PMC2588488

[7] V. Gradinaru., Molecular and cellular approaches for diversifying and extending optogenetics. Cell 141, 154–165 (2010). 20303157 10.1016/j.cell.2010.02.037 PMC4160532

[8] C. Zhang., Optimized photo-stimulation of halorhodopsin for long-term neuronal inhibition. BMC Biol. 17, 95 (2019). 31775747 10.1186/s12915-019-0717-6 PMC6882325

[9] M. Andersson., Structural dynamics of light-driven proton pumps. Structure 17, 1265–1275 (2009). 19748347 10.1016/j.str.2009.07.007

[10] C. Wickstrand, R. Dods, A. Royant, R. Neutze, Bacteriorhodopsin: Would the real structural intermediates please stand up? Biochim. Biophys. Acta 1850, 536–553 (2015). 24918316 10.1016/j.bbagen.2014.05.021

[11] C. Punwong, T. J. Martínez, S. Hannongbua, Direct QM/MM simulation of photoexcitation dynamics in bacteriorhodopsin and halorhodopsin. Chem. Phys. Lett. 610–611, 213–218 (2014).

[12] J. K. Lanyi, Bacteriorhodopsin. Annu. Rev. Physiol. 66, 665–688 (2004). 14977418 10.1146/annurev.physiol.66.032102.150049

[13] E. Nango., A three-dimensional movie of structural changes in bacteriorhodopsin. Science 354, 1552–1557 (2016). 28008064 10.1126/science.aah3497

[14] P. Nogly., Retinal isomerization in bacteriorhodopsin captured by a femtosecond x-ray laser. Science 361, eaat0094 (2018). 29903883 10.1126/science.aat0094

[15] G. Nass Kovacs., Three-dimensional view of ultrafast dynamics in photoexcited bacteriorhodopsin. Nat. Commun. 10, 3177 (2019). 31320619 10.1038/s41467-019-10758-0 PMC6639342

[16] P. Skopintsev., Femtosecond-to-millisecond structural changes in a light-driven sodium pump. Nature 583, 314–318 (2020). 32499654 10.1038/s41586-020-2307-8

[17] J. Sasaki., Conversion of bacteriorhodopsin into a chloride ion pump. Science 269, 73–75 (1995). 7604281 10.1126/science.7604281

[18] T. Hasemi, T. Kikukawa, N. Kamo, M. Demura, Characterization of a cyanobacterial chloride-pumping rhodopsin and its conversion into a proton pump. J. Biol. Chem. 291, 355–362 (2016). 26578511 10.1074/jbc.M115.688614 PMC4697170

[19] S. Yoshizawa., Functional characterization of flavobacteria rhodopsins reveals a unique class of light-driven chloride pump in bacteria. Proc. Natl. Acad. Sci. U.S.A. 111, 6732–6737 (2014). 24706784 10.1073/pnas.1403051111 PMC4020065

[20] K. Kim., Crystal structure and functional characterization of a light-driven chloride pump having an NTQ motif. Nat. Commun. 7, 12677 (2016). 27554809 10.1038/ncomms12677 PMC4999514

[21] J. H. Yun., Non-cryogenic structure of a chloride pump provides crucial clues to temperature-dependent channel transport efficiency. J. Biol. Chem. 294, 794–804 (2019). 30455349 10.1074/jbc.RA118.004038 PMC6341376

[22] J. H. Yun., Pumping mechanism of NM-R3, a light-driven bacterial chloride importer in the rhodopsin family. Sci. Adv. 6, eaay2042 (2020). 32083178 10.1126/sciadv.aay2042 PMC7007266

[23] M. Schmidt, S. Rajagopal, Z. Ren, K. Moffat, Application of singular value decomposition to the analysis of time-resolved macromolecular x-ray data. Biophys. J. 84, 2112–2129 (2003). 12609912 10.1016/S0006-3495(03)75018-8 PMC1302779

[24] F. Schotte., Watching a signaling protein function in real time via 100-ps time-resolved Laue crystallography. Proc. Natl. Acad. Sci. U.S.A. 109, 19256–19261 (2012). 23132943 10.1073/pnas.1210938109 PMC3511082

[25] Y. O. Jung., Volume-conserving trans-cis isomerization pathways in photoactive yellow protein visualized by picosecond X-ray crystallography. Nat. Chem. 5, 212–220 (2013). 23422563 10.1038/nchem.1565 PMC3579544

[26] F. Zhang., The microbial opsin family of optogenetic tools. Cell 147, 1446–1457 (2011). 22196724 10.1016/j.cell.2011.12.004 PMC4166436

[27] S. Boutet, G. J. Williams, The coherent X-ray imaging (CXI) instrument at the linac coherent light source (LCLS). New J. Phys. 12, 035024 (2010).

[28] M. Liang., The coherent X-ray imaging instrument at the linac coherent light source. J. Synchrotron Radiat. 22, 514–519 (2015). 25931062 10.1107/S160057751500449X PMC4416669

[29] U. Weierstall., Lipidic cubic phase injector facilitates membrane protein serial femtosecond crystallography. Nat. Commun. 5, 3309 (2014). 24525480 10.1038/ncomms4309 PMC4061911

[30] P. Hart., The CSPAD megapixel x-ray camera at LCLS. Proc. SPIE 8504, X-Ray Free-Electron Lasers: Beam Diagnostics, Beamline Instrumentation, and Applications, 85040C, 10.1117/12.930924 (2012).

[31] A. Barty., *Cheetah*: Software for high-throughput reduction and analysis of serial femtosecond X-ray diffraction data. J. Appl. Cryst. 47, 1118–1131 (2014). 24904246 10.1107/S1600576714007626 PMC4038800

[32] X. Li, C. Li, H. Liu, *ClickX*: A visualization-based program for preprocessing of serial crystallography data. J. Appl. Cryst. 52, 674–682 (2019). 31236097 10.1107/S1600576719005363 PMC6557179

[33] M. Harmand., Achieving few-femtosecond time-sorting at hard X-ray free-electron lasers. Nat. Photonics 7, 215–218 (2013).

[34] T. A. White., CrystFEL: A software suite for snapshot serial crystallography. J. Appl. Cryst. 45, 335–341 (2012).

[35] Z. Ren., Laue crystallography: Coming of age. J. Synchrotron Radiat. 6, 891–917 (1999).

[36] J. Tenboer., Time-resolved serial crystallography captures high-resolution intermediates of photoactive yellow protein. Science 346, 1242–1246 (2014). 25477465 10.1126/science.1259357 PMC4361027

[37] K. Pande., Femtosecond structural dynamics drives the trans/cis isomerization in photoactive yellow protein. Science 352, 725–729 (2016). 27151871 10.1126/science.aad5081 PMC5291079

[38] M. Schmidt, Time-resolved macromolecular crystallography at pulsed X-ray sources. Int. J. Mol. Sci. 20, 1401 (2019). 10.3390/ijms20061401 PMC6470897 30897736

[39] S. Pandey., Time-resolved serial femtosecond crystallography at the European XFEL. Nat. Methods 17, 73–78 (2020). 31740816 10.1038/s41592-019-0628-z PMC9113060

[40] T. C. Terwilliger, J. Berendzen, Bayesian difference refinement. Acta Crystallogr. D Biol. Crystallogr. 52, 1004–1011 (1996). 15299610 10.1107/S0907444996006725

[41] M. D. Winn., Overview of the *CCP*4 suite and current developments. Acta Crystallogr. D Biol. Crystallogr. 67, 235–242 (2011). 21460441 10.1107/S0907444910045749 PMC3069738

[42] P. Emsley, B. Lohkamp, W. G. Scott, K. Cowtan, Features and development of Coot. Acta Crystallogr. D Biol. Crystallogr. 66, 486–501 (2010). 20383002 10.1107/S0907444910007493 PMC2852313

[43] N. W. Moriarty, R. W. Grosse-Kunstleve, P. D. Adams, Electronic Ligand Builder and Optimization Workbench (eLBOW): a tool for ligand coordinate and restraint generation. Acta Crystallogr. D Biol. Crystallogr. 65, 1074–1080 (2009). 19770504 10.1107/S0907444909029436 PMC2748967

[44] P. D. Adams., PHENIX: A comprehensive python-based system for macromolecular structure solution. Acta Crystallogr. D Biol. Crystallogr. 66, 213–221 (2010). 20124702 10.1107/S0907444909052925 PMC2815670

[45] E. F. Pettersen., UCSF Chimera–A visualization system for exploratory research and analysis. J. Comput. Chem. 25, 1605–1612 (2004). 15264254 10.1002/jcc.20084

[46] L. Schrödinger, The PyMOL Molecular Graphics System, (Version 2.2, 2015). Schrödinger, LLC.

[47] J. E. J. Mills, P. M. Dean, Three-dimensional hydrogen-bond geometry and probability information from a crystal survey. J. Comput. Aided Mol. Des. 10, 607–622 (1996). 9007693 10.1007/BF00134183

[48] M. A. Lomize, I. D. Pogozheva, H. Joo, H. I. Mosberg, A. L. Lomize, OPM database and PPM web server: Resources for positioning of proteins in membranes. Nucleic Acids Res. 40, D370–D376 (2012). 21890895 10.1093/nar/gkr703 PMC3245162

[49] J. Lee., CHARMM-GUI input generator for NAMD, GROMACS, AMBER, OpenMM, and CHARMM/OpenMM simulations using the CHARMM36 additive force field. J. Chem. Theory Comput. 12, 405–413 (2016). 26631602 10.1021/acs.jctc.5b00935 PMC4712441

[50] R. B. Best., Optimization of the additive CHARMM all-atom protein force field targeting improved sampling of the backbone φ, ψ and side-chain χ(1) and χ(2) dihedral angles. J. Chem. Theory Comput. 8, 3257–3273 (2012). 23341755 10.1021/ct300400x PMC3549273

[51] M. J. Abraham., Gromacs: High performance molecular simulations through multi-level parallelism from laptops to supercomputers. SoftwareX 1–2, 19–25 (2015).

[52] W. Humphrey, A. Dalke, K. Schulten, VMD: Visual molecular dynamics. J. Mol. Graph. 14, 33–38, 27–28 (1996). 8744570 10.1016/0263-7855(96)00018-5

[53] T. Arlt, S. Schmidt, W. Zinth, U. Haupts, D. Oesterhelt, The initial reaction dynamics of the light-driven chloride pump halorhodopsin. Chem. Phys. Lett. 241, 559–565 (1995).

[54] S. Tahara., Ultrafast photoreaction dynamics of a light-driven sodium-ion-pumping retinal protein from Krokinobacter eikastus revealed by femtosecond time-resolved absorption spectroscopy. J. Phys. Chem. Lett. 6, 4481–4486 (2015). 26582475 10.1021/acs.jpclett.5b01994

[55] I. H. M. van Stokkum, D. S. Larsen, R. van Grondelle, Global and target analysis of time-resolved spectra. Biochim. Biophys. Acta 1657, 82–104 (2004). 15238266 10.1016/j.bbabio.2004.04.011

[56] H. Liu, ClR data from time-resolved serial-femtosecond-crystallography. GitHub. https://github.com/LiuLab-CSRC/Clr-Dynamics. Deposited 15 December 2020.

