# Supplementary material for: Early-stage dynamics of chloride ion–pumping rhodopsin revealed by a femtosecond X-ray laser
Source: Proc Natl Acad Sci U S A. 2021 Mar 22;118(13):e2020486118. doi: 10.1073/pnas.2020486118 (PMC8020794; doi:10.1073/pnas.2020486118)
Supplement: Supplementary File [file pnas.2020486118.sapp.pdf]

PNAS
www.pnas.org

Supplementary Information for

Early-stage dynamics of chloride ion pumping rhodopsin revealed by femtosecond X-ray laser

Authors: Ji-Hye Yun[1,†], Xuanxuan Li[2,3,†], Jianing Yue[4], Jae-Hyun Park[1], Zeyu Jin[1], Chufeng Li[5], Hao Hu[5], Yingchen Shi[2,3], Suraj Pandey[6], Sergio Carbajo[7], Sébastien Boutet[7], Mark S. Hunter[7], Mengning Liang[7], Raymond G. Sierra[7], Thomas J. Lane[7], Liang Zhou[4], Uwe Weierstall[5], Nadia A Zatsepin[5,8], Mio Ohki[9], Jeremy R. H. Tame[9], Sam-Yong Park[9], John C. H. Spence[5], Wenkai Zhang[4], Marius Schmidt[6*], Weontae Lee[1*] and Haiguang Liu[2,4*]

Affiliations:
[1]Department of Biochemistry, College of Life Sciences & Biotechnology, Yonsei University, 50 Yonsei-ro, Seodaemun-gu, Seoul, 120-749, South Korea
[2]Complex Systems Division, Beijing Computational Science Research Center, 8 E Xibeiwang Rd, Haidian, Beijing, 100193, People's Republic of China
[3]Department of Engineering Physics, Tsinghua University, Beijing, 100086, People's Republic of China
[4]Physics Department, Beijing Normal University, No. 19 Xinjiekouwai St., Haidian, Beijing, 100875, People's Republic of China
[5]Department of Physics, Arizona State University, Box 871504, Tempe, AZ, 85287, USA
[6]Physics Department, University of Wisconsin, Milwaukee, 3135 North Maryland Ave, Milwaukee, Wisconsin, 53201, USA
[7]Linac Coherent Light Source, SLAC National Accelerator Laboratory, 2575 Sand Hill Road, Menlo Park, CA, 94025, United States
[8]ARC Centre of Excellence in Advanced Molecular Imaging, Department of Chemistry and Physics, La Trobe Institute for Molecular Science, La Trobe University, Melbourne, Victoria 3086, Australia
[9]Drug Design Laboratory, Graduated School of Medical Life Science, Yokohama City University, Suehiro 1-7-29 Tsurumi-ku, Yokohama 230-0045, Japan

*Correspondence authors: m-schmidt@uwm.edu (M.S.); wlee@spin.yonsei.ac.kr (W.L.); hgliu@csrc.ac.cn (H.L.)

†These authors contributed equally to this work.

This PDF file includes:

Figures S1 to S12
Tables S1 to S3
Legend for Movies S1

Other supplementary materials for this manuscript include the following:

Movies S1

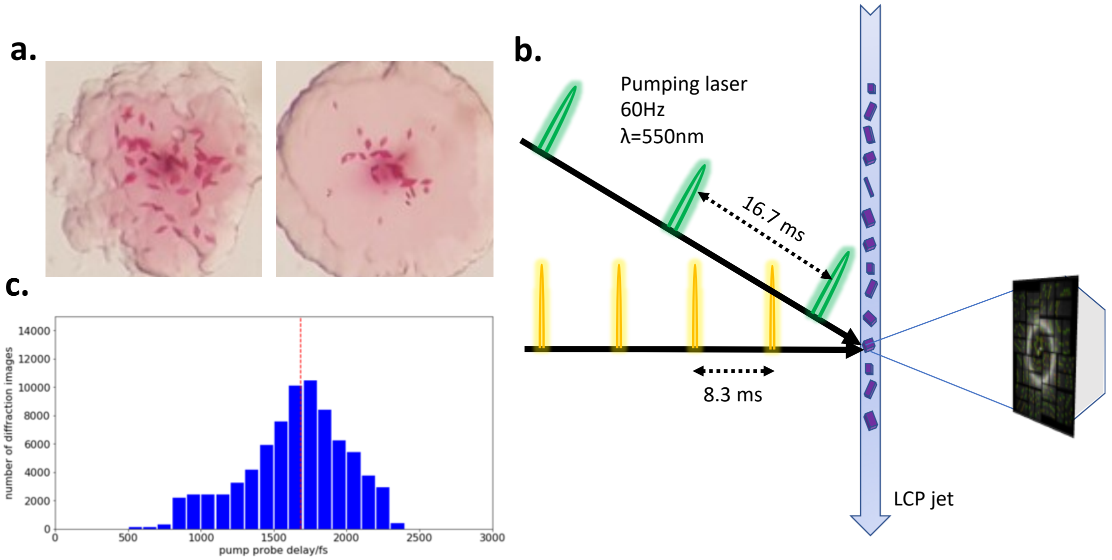

Fig. S1. ClR crystal samples, experimental setup and time binning. (**a**) ClR microcrystals in LCP, with and the crystals are observable in purple color. (**b**) Experimental setup, the femtosecond (~100 fs, FWHM) pump laser λ=550 nm was operating at 60 Hz, resulting an on-off interleaved pumping scheme. LCP injector was used to deliver microcrystals to the pump-laser/XFEL interception point. (**c**) The time tool was used for fine binning the diffraction patterns collected at nominal time delay Δt=1 ps. The red dotted line indicate the binning boundary between the two sub-datasets, with averaged time delays of 1 ps and 2 ps, respectively.

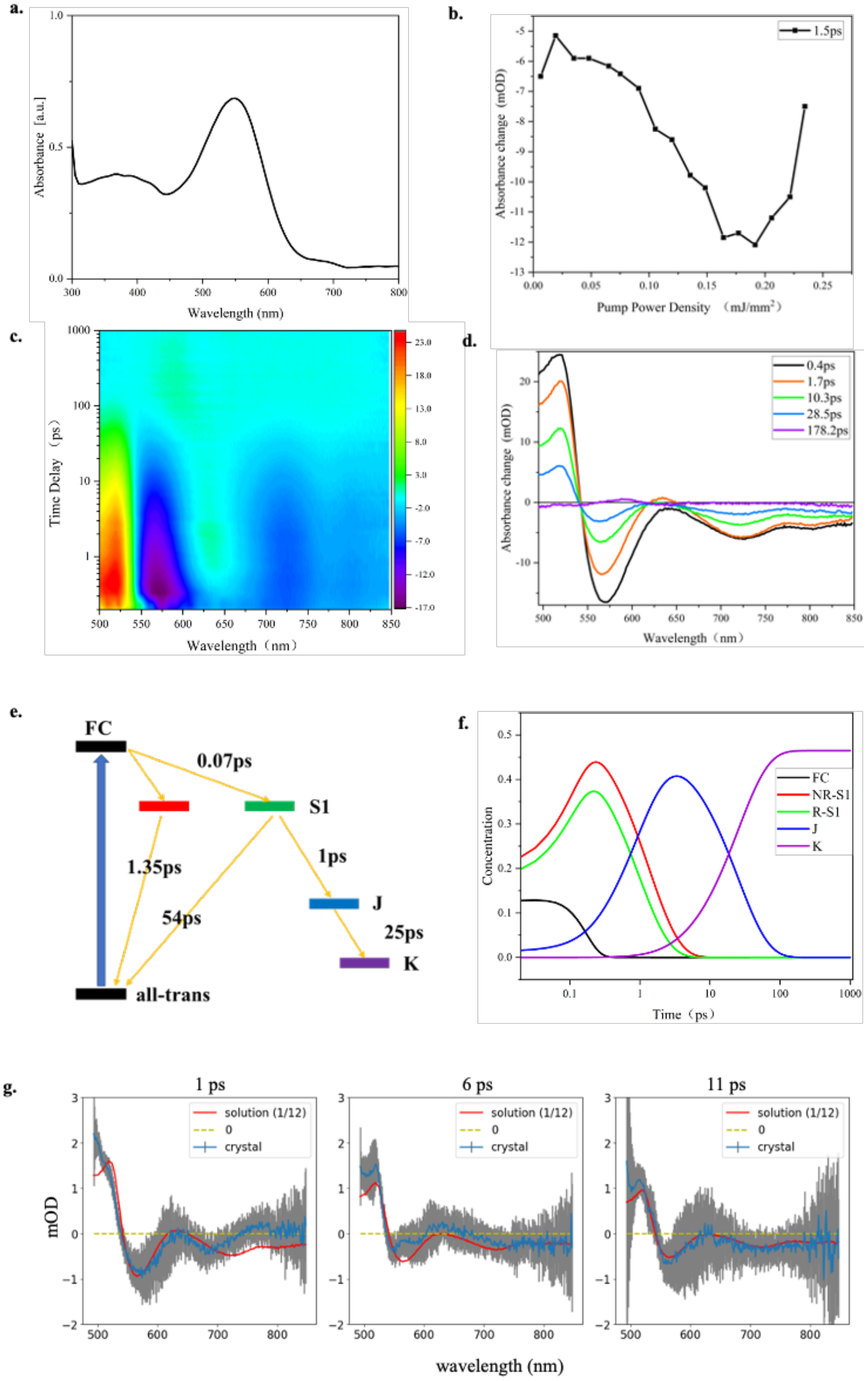
a.
Absorbance [a.u.]
Wavelength (nm)
b.
1.5ps
Absorbance change (mOD)
Pump Power Density (mJ/mm²)
c.
Time Delay (ps)
Wavelength (nm)
d.
0.4ps
1.7ps
10.3ps
28.5ps
178.2ps
Absorbance change (mOD)
Wavelength (nm)
e.
FC
0.07ps
S1
1ps
1.35ps
J
54ps
25ps
K
all-trans
f.
FC
NR-S1
R-S1
J
K
Concentration
Time (ps)
g.
1 ps
6 ps
11 ps
solution (1/12)
0
crystal
mOD
wavelength (nm)

Fig. S2. Spectroscopy data and kinetics analysis. (**a**) UV-Vis spectrum for ClR solution in dark state. (**b**) Pumping laser power titration result. The transient absorption signals at 1.5 ps and wavelength range between 580 and 620 nm. The linear regime is up to 0.16 mJ/mm^2. (**c**) Time-resolved UV-Vis absorption spectra within 1000 ps after light activation. (**d**) The spectra from 500 nm to 850 nm at five representative time delays. (**e**) Schematic drawing of the kinetics model for photoexcited reactions. The model is based on recent studies (see main text), with the state labels have the following meaning: all-trans, retinal configuration in the dark state; FC, Frank-Condon state after photon absorption; S1, excited electronic state, also denoted as I in photocycles; J, 13-cis J-intermediate that is vibrationally hot; and K, relaxed 13-cis state. There is a branching from S1 state, which can either return to all-trans state (NR-S1) or proceed to isomerization (R-S1). (**f**) The time evolution of the species in the kinetics model shown in (**e**). The progression of the light triggered reactions shows the fractions of components within 1000 ps. From 1 ps to 10 ps, the J-state ClR dominates the species, while the population shifts to K-state from 50 ps to 100 ps. (**g**) Comparison of TAS data of ClR solution and ClR crystals. The signals from ClR solution was scaled down by 12 times for all three time delays to match the levels of ClR crystal signals. The crystal ClR signals were averaged over 20 measurements and the shaded region marks the corresponding standard deviations.

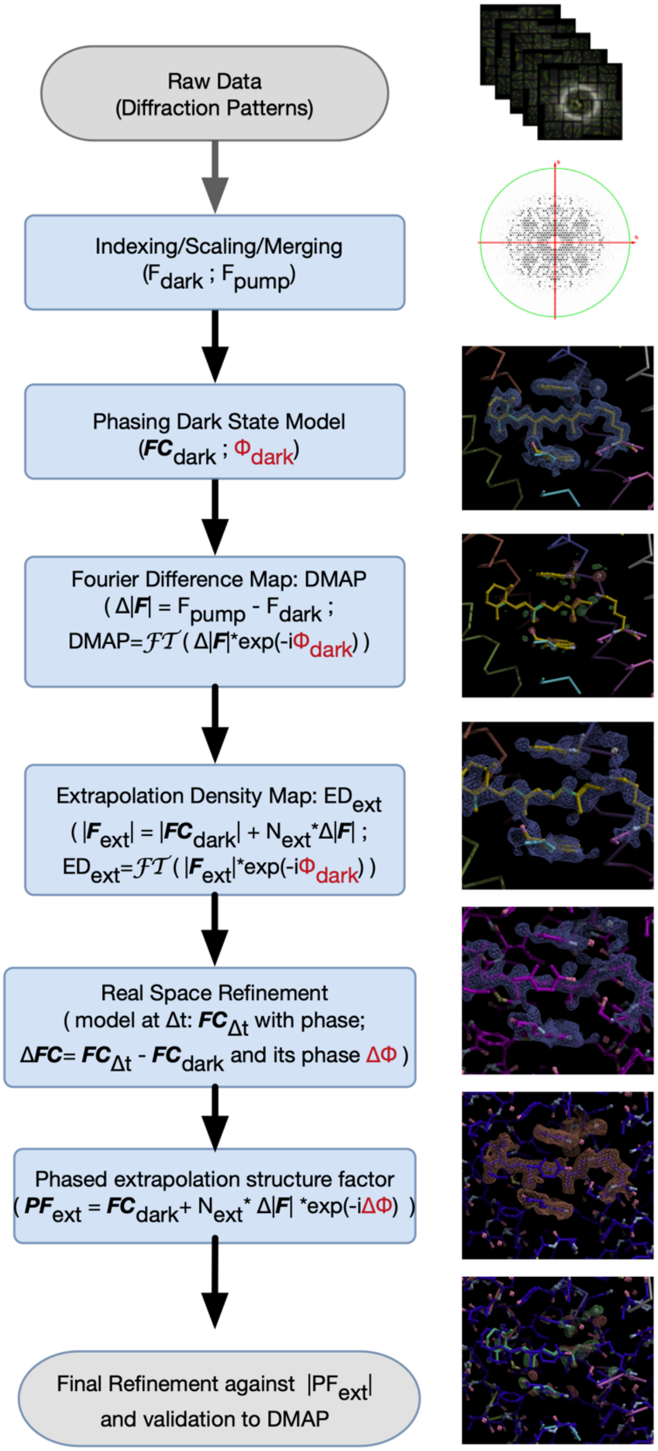

Fig. S3. Flowchart for data analysis and interpretation. The pipeline summarizes the workflow, including raw data reduction, Fourier difference density map calculation, composing extrapolated structure factors and extrapolated map, and structure refinements. Details are referred to Method section in main text.

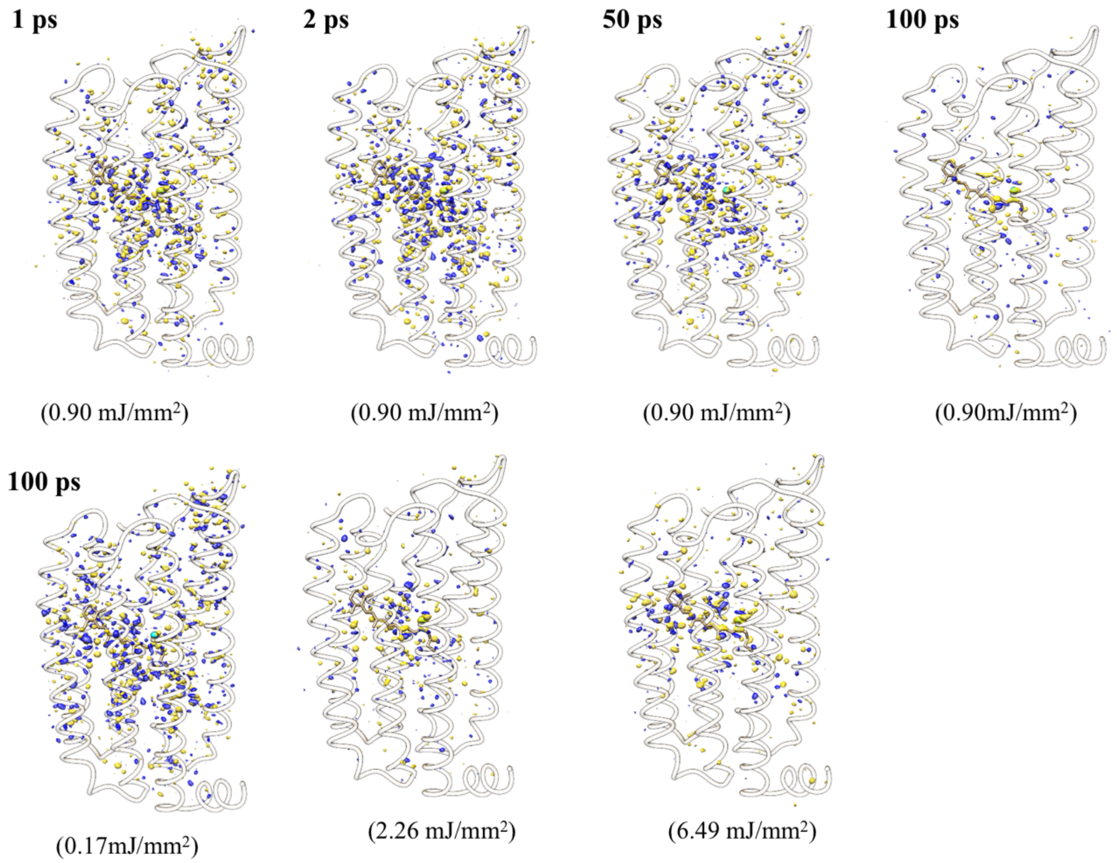

Fig. S4. The observed difference maps between the pump and dark datasets. The pump laser power level for 1,2,50 ps time delays was 0.90 mJ/mm^2; the time delay Δt=100 ps was measured at four power levels (0.17 mJ/mm^2 – 6.49 mJ/mm^2). The difference features are shown at contour level of 3.5 σ, with gold for negative and blue for positive features. Note that the 100 ps dataset measured at 0.17 mJ/mm^2 pumping power is noisy, and the features near the retinal are weaker compared to other datasets. The measured amplitude difference $|\Delta F_{obs}|$ and the dark state phase φ_{dark} were used to calculate the difference maps.

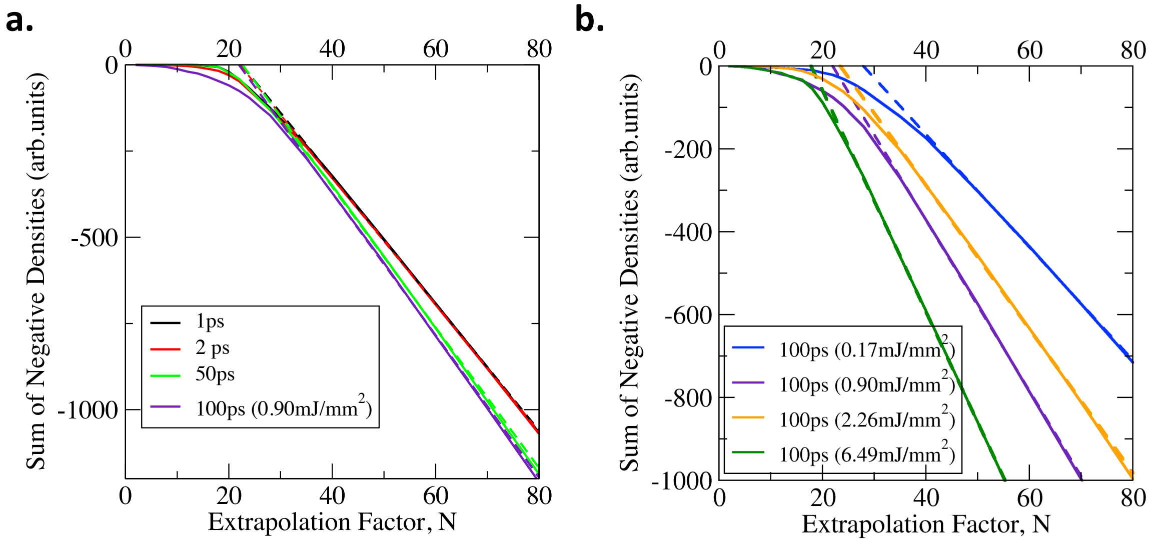

Fig. S5. The determination of extrapolation factors. (**a**) The negative densities in the extrapolated maps centered at C13,C14 and $Cl^{-}1$ atoms are plotted against the extrapolation factors. The diverging points indicate the best estimated extrapolation factors, beyond which the negative density linearly increases. The dashed lines show the fitting results of the linear regime. (**b**) the extrapolation factor estimation for the datasets collected at Δt=100 ps with 4 power levels of the pump laser.

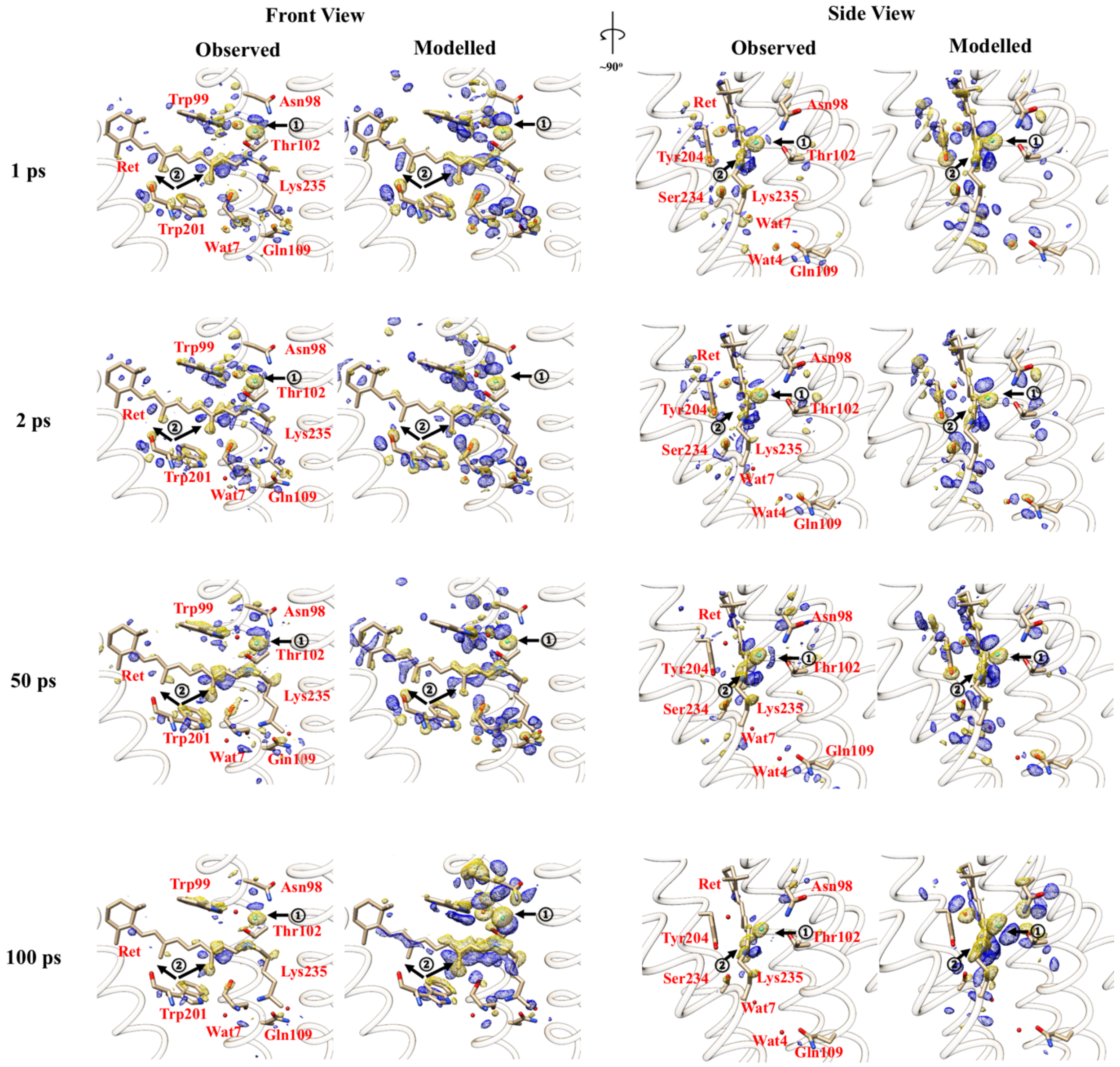

Fig. S6. The Fourier difference density maps at four time delays. The signal levels for major peaks are summarized in Table S2. The isomerization of retinal and the movement of Cl^- ion near the Schiff base are evident in the difference maps. The front view is shown in the two left columns, with the observed DMAPs and model derived DMAPs organized side-by-side for direct comparison. The two right columns show a side view, approximately perpendicular to the front view. The observed DMAPs are shown at 3.5 σ contour level, while the calculated difference maps between structures at Δt and the dark state structure are shown at a contour level of 7.0 σ, to match the signal features in the observed difference maps. Important residues are labeled and the prominent features are indicated with black arrows. In all maps, negative and positive densities are colored in gold and blue respectively. The observed DMAPs are based on measured amplitude difference $|\Delta F_{obs}|$ and the dark state phase φ_{dark}; the modelled DMAPs are calculated from $\Delta\mathbf{F}_{model}=\mathbf{F}_{model,pump} - \mathbf{F}_{model,dark}$.

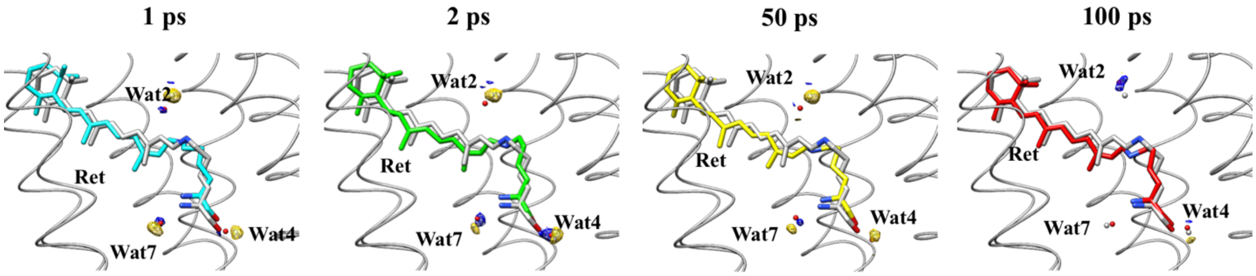

Fig. S7. The Fourier difference density maps near three water molecules at four time delays. Difference maps are contoured at +/– 2.5 σ levels in gold/blue for negative/positive densities. The retinal and three water molecules are shown on top of the backbone of dark state structure. The dark state retinal and water molecules are shown in gray color as references. The observed DMAPs are based on measured amplitude difference $|\Delta F_{obs}|$ and the dark state phase φ_{dark}.

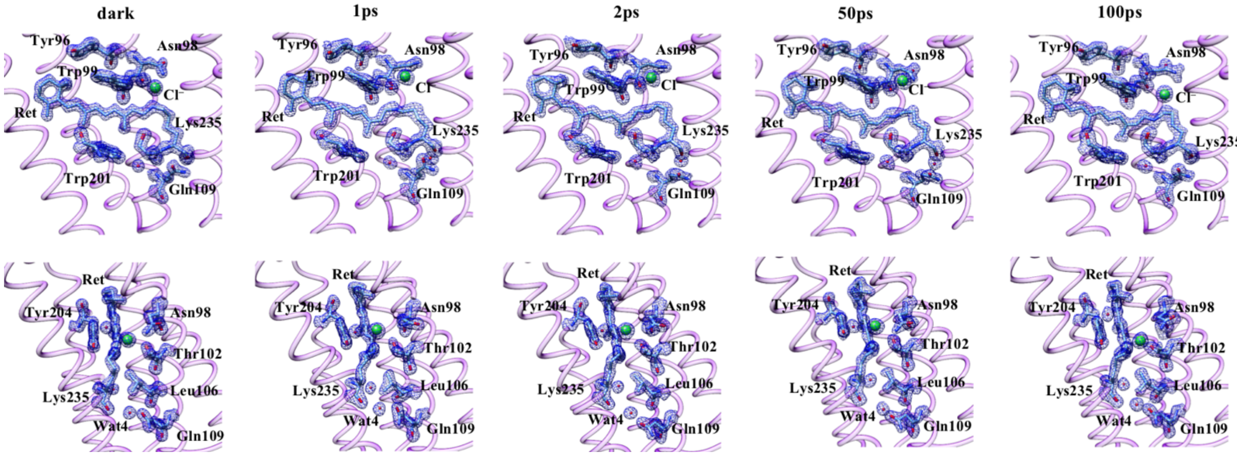

Fig. S8. The refined structures and the electron density maps. The electron density maps (2Fo-mFc) are shown at 2 σ contour level for atoms near the retinal in two orthogonal views. The structures at each time delay are refined against the phase-extrapolated structure factors, $|\mathbf{PF}_{ext}|$ where $\mathbf{PF}_{ext} = \mathbf{FC}_{dark} + N_{ext} \times \Delta\mathbf{F}_{obs}$. The dark state structure is shown in gray color as a reference to indicate the structural changes at each time delay.

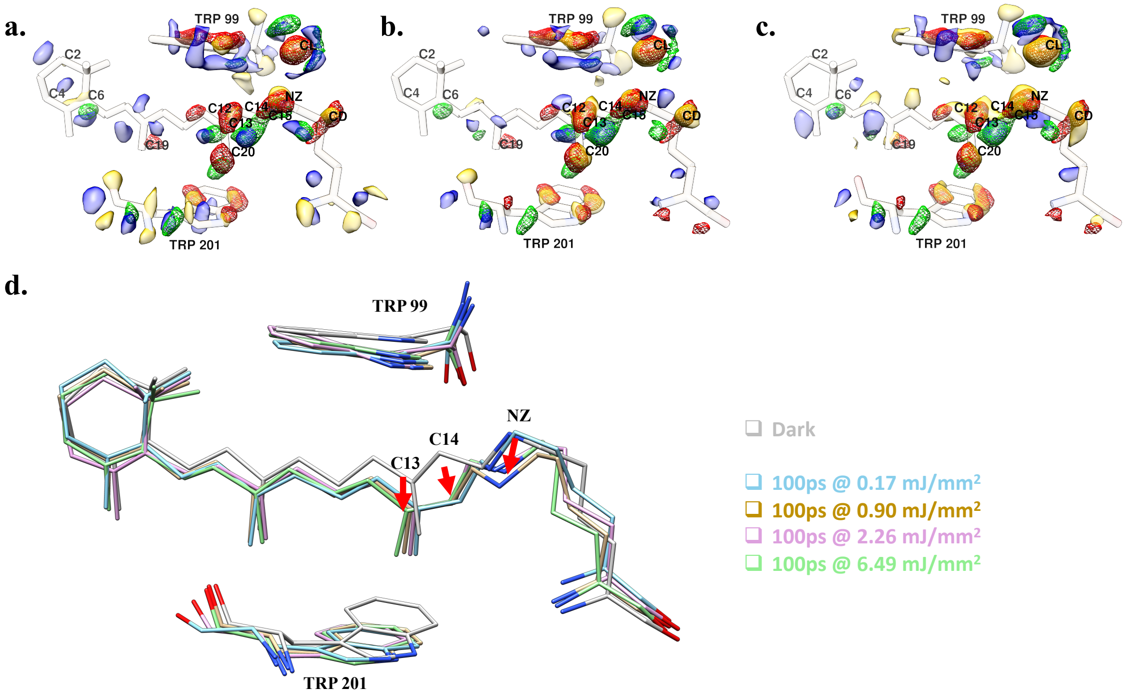

Fig. S9. The power influence on the difference density map signals. The DMAPs between the 100 ps and dark state are shown at 3σ levels near the retinal. The major peaks centered around the Cl^{-}1, C13, C14, Nζ, are consistent in the four pump laser power levels: (**a**) 0.17mJ/mm^2, (**b**) 2.26 mJ/mm^2, (**c**) 6.49 mJ/mm^2, the DMAPs are shown in surface representation (yellow=-3σ; blue=+3σ); the DMAP for laser power level of 0.90 mJ/mm^2 is shown in meshed representation (red=-3σ; green=+3σ) as the reference for comparison. (**d**) The refined models compared to the dark structure (gray color), the major atomic displacements are indicated with red arrows. The observed DMAPs are based on measured amplitude difference $|\Delta F_{obs}|$ and the dark state phase φ_{dark}.

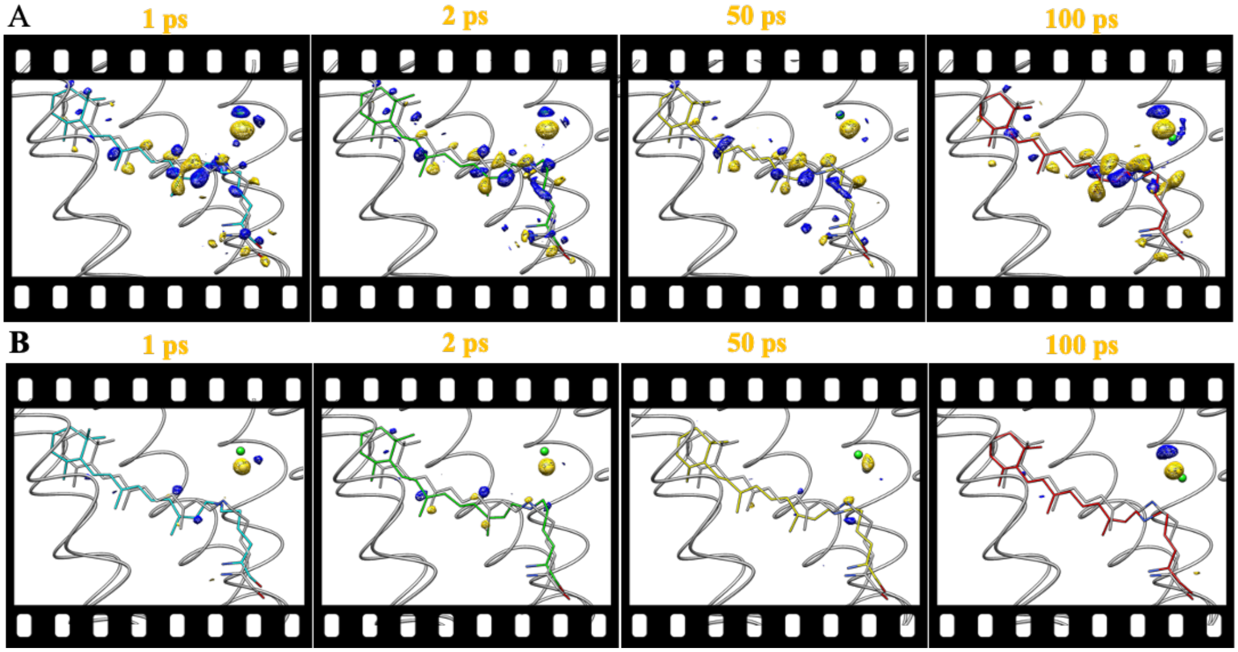

Fig. S10. Signals observed in the difference electron density maps are mostly explained by refined models. (**A**) The observed DMAPs showing electron density differences around the retinal and the nearby chloride ion. The densities are contoured at +/– 3.5 σ for 1,2,50 ps time delays; and +/– 3.0 σ for 100 ps time delay. Positive and negative densities are colored in blue and gold respectively. (**B**) The ΔDMAPs between $DMAP_{obs}$ and $DMAP_{model}$ reveal the reduction in difference signals after subtracting the modelled differences ($DMAP_{model}$) computed between the activated structures and the dark state structure. The contour levels and coloring scheme are the same as in (A). The observed electron density differences near the retinal are significantly reduced, indicating that refined structures capture the associated conformation changes. At 100 ps, there are substantial signals of Cl^- close to Trp99, suggesting that the Cl^- in some activated ClR proteins have not migrated towards Thr102. This is consistent with the diffusive movement of Cl^- after dissociation from the binding site in the dark state.

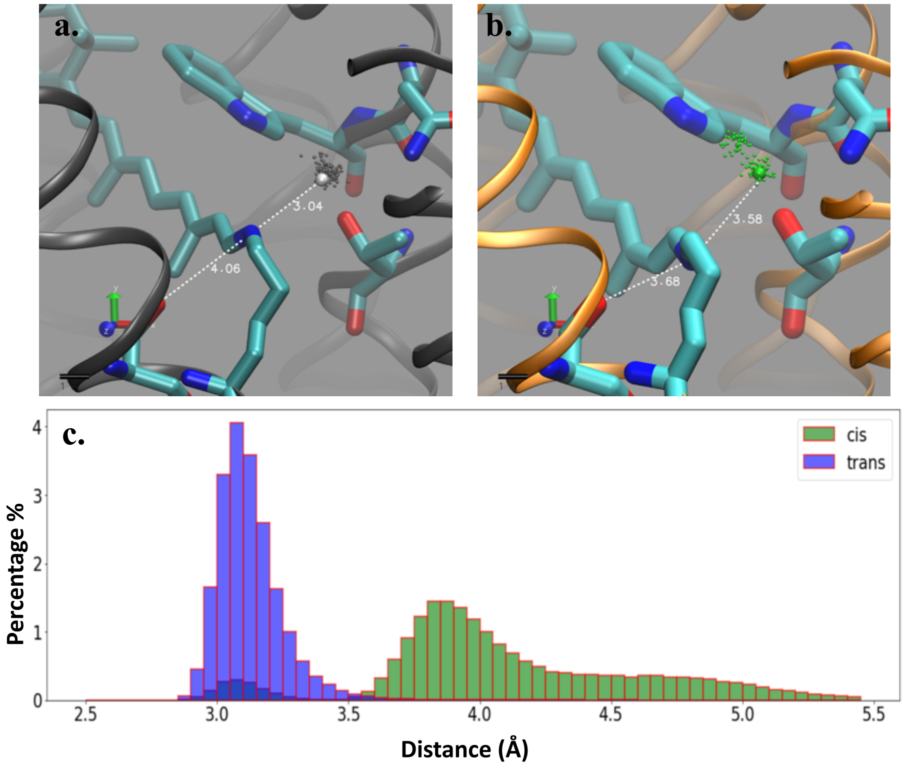

Fig. S11. The positions of Cl^- depend on the retinal configurations. (**a**) The *all-trans* retinal stabilizes the Cl^- as the Schiff base forms stable attractive interactions with the Cl^- ion. (**b**) The Cl^- moves towards the Trp99 in the case of the *13-cis* retinal. The distance between Oγ atom of the Ser234 and the Schiff Base is shorter in the case of *13-cis* retinal. (**c**) the distributions of distances between Cl^- and the Schiff base observed in molecular dynamics simulations.

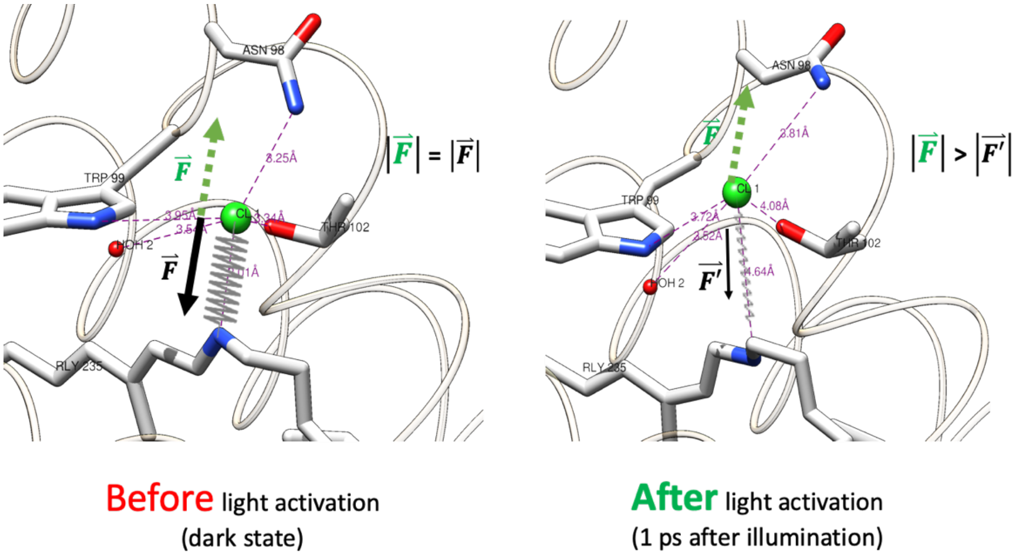

Figure S12. Schematic explanation of Cl^- dissociation upon retinal isomerization. The force exerted on the Cl^- from the protonated Schiff base is weakened due to the isomerization, as a result, the Cl- ion is pulled upward away from the PSB.

Table S1.

Statistics of data collections and structure Refinement.

Statistics for Data Collection

Dataset	Dark	1ps	2ps	50ps	100ps-power1	100ps-power2	100ps-power3	100ps-power4
Pumping Power (GW/mm^2)	N/A	9.0	9.0	9.0	9.0	1.7	26.3	64.9
Space group	C2							
Unit cell [a, b, c (Å), β(°)]	103.37, 50.09, 69.39, 109.69							
No. of hits	58,194	39,333	39,332	36,944	57,447	11,6021	38,995	78,978
No. of indexed patterns	37,044	29,469	25,690	23,718	35,106	47,795	22,474	41,253
Indexing rate (%)	63.7	74.9	65.3	64.2	61.1	41.2	57.6	53.2
Resolution (Å)[#]	20.90-1.65 (1.71-1.65)	20.90-1.73 (1.76-1.73)	20.90-1.72 (1.75-1.72)	20.90-1.77 (1.80-1.77)	20.90-1.74 (1.77-1.74)	20.90-1.74 (1.77-1.74)	20.90-1.77 (1.80-1.77)	20.90-1.81 (1.84-1.81)
No. of total reflections	15269853 (137346)	10823779 (116435)	10876489 (115252)	9616447 (118058)	14111488 (118719)	20758716 (254974)	8864522 (108059)	16107548 (157670)
No. of unique reflections	36756 (1783)	35361 (1736)	35998 (1783)	33081 (1709)	34776 (1694)	34776 (1694)	33081 (1709)	30942 (1535)
Rsplit (%)	13.37 (67.98)	14.44 (65.11)	13.90 (64.27)	14.98 (62.61)	13.54 (64.93)	10.73 (63.13)	14.44 (68.97)	11.14 (64.31)
SNR	5.52 (1.58)	5.17 (1.55)	5.26 (1.54)	4.86 (1.56)	5.37 (1.60)	6.65 (1.60)	5.17 (1.54)	6.37 (1.59)
Completeness (%)	100 (100)	100 (100)	100 (100)	100 (100)	100 (100)	100 (100)	100 (100)	100 (100)
Multiplicity	424.2 (77.0)	306.1 (67.1)	302.1 (64.6)	290.7 (69.1)	405.8 (70.1)	596.9 (150.5)	268.0 (63.2)	520.6 (102.7)
CC1/2	0.9838 (0.6769)	0.9767 (0.7035)	0.9778 (0.7030)	0.9767 (0.7324)	0.9829 (0.6863)	0.9878 (0.7159)	0.9786 (0.6918)	0.9890 (0.7489)
CC*	0.9959 (0.8985)	0.9941 (0.9088)	0.9943 (0.9086)	0.9941 (0.9195)	0.9957 (0.9022)	0.9969 (0.9134)	0.9945 (0.9043)	0.9972 (0.9254)

[#] The numbers in the parentheses correspond to statistics of the highest resolution shell. These are the statistics for the raw data by treating the datasets as conventional crystallography data.

Statistics for Model Refinement

Dataset	Dark	1ps	2ps	50ps	100ps-power1	100ps-power2	100ps-power3	100ps-power4
**Pumping Power (GW/mm^2)**	N/A	9.0	9.0	9.0	9.0	1.7	26.3	64.9
Extrapolation Factor	N/A	22	22	22	22	28	22	18
**Resolution (Å)**	20.15 - 1.65 (1.709 - 1.65)	20.15 - 1.85 (1.916 - 1.85)	20.15 - 1.85 (1.916 - 1.85)	20.15 - 1.85 (1.916 - 1.85)	20.15 - 1.85 (1.196 – 1.85)	20.15 - 1.85 (1.916 - 1.85)	20.15 - 1.85 (1.916 - 1.85)	20.15 - 1.85 (1.916 - 1.85)
**No. of reflections**	36964 (3001)	27267 (2491)	27264 (2491)	27267 (2491)	27266 (2491)	27267 (2491)	27268 (2491)	27268 (2491)
**No. of reflections for Rfree**	1829 (154)	1373 (122)	1372 (122)	1372 (122)	1372 (122)	1373 (122)	1373 (122)	1373 (122)
**Rwork (%)#**	17.37 (30.70)	27.66 (39.21)	27.83 (40.58)	28.03 (39.46)	25.26 (38.69)	35.65 (48.44)	25.85 (40.00)	25.78 (36.38)
**Rfree (%)**	19.52 (32.31)	33.63 (45.01)	32.95 (40.82)	33.09 (43.85)	30.06 (41.97)	40.58 (45.84)	30.73 (47.08)	32.09 (43.25)
**RMS bonds**	0.018	0.025	0.023	0.023	0.023	0.013	0.008	0.024
**RMS angles**	1.51	2.47	2.42	2.27	2.20	1.69	1.26	2.25
**No. of atoms [Protein]**	2,072	2,072	2,072	2,072	2,072	2,072	2,072	2,072
**No. of atoms [Ligands]**	121	121	121	121	121	121	121	121
**No. of atoms [Solvent]**	106	106	106	106	106	106	106	106
**Ramachandran plot[Favored](%)**	98.47	91.22	87.40	93.13	93.13	87.40	96.18	96.18
**Ramachandran plot[Allowed](%)**	1.53	7.25	9.92	6.49	6.87	9.92	3.82	3.05
**Ramachandran plot[Disallowed](%)**	0.00	1.53	2.67	0.38	0.00	2.67	0.00	0.76

The structures are refined against the amplitudes of extrapolated structure factors with phase correction. The structure factor is obtained via extrapolation method with phase correction: $\mathbf{PF}_{ext} = \mathbf{FC}_{dark} + N_{ext} \times \Delta\mathbf{F}_{obs}$, where $\mathbf{FC}_{dark}$ and $\Delta\mathbf{F}_{obs}$ are the calculated structure factor from dark state model and the observed difference structure factors, both include amplitudes and phases. $\Delta\mathbf{F}_{obs} = |\Delta\mathbf{F}_{obs}| e^{-i\phi\Delta}$ ($|\Delta\mathbf{F}_{obs}|$ is the experimental amplitude, and the ϕ_{Δ} is the computed phase between the dark model and the real-space refined pumped model)

Table S2. Significant peak information in observed DMAPs

peaks	1ps	2ps	50ps	100ps (0.17mJ/mm^2)	100ps (0.90mJ/mm^2)	100ps (2.63mJ/mm^2)	100ps (6.49mJ/mm^2)
Cl1(-)	-14.4	-13.5	-10.5	-8.7	-8.9	-12.0	-12.4
Cl1(+1)	5.6	4.7	4.0	4.1	4.2	5.0	5.2
Cl1(+2)	4.5	3.8	3.7	3.9	4.1	3.6	4.1
Cl1(+3)	NA	NA	3.8	4.7	4.2	3.5	connected with Cl1(+2)
C12(-)	-5.0	-6.0	-4.9	-4.4	-3.1	-3.5	-5.4
C12(+)	5.4	5.2	5.8	4.9	3.8	4.5	3
C14(-)	-6.2	-6.1	-6.4	-4.1	-4.9	-6.6	-5.7
C14(+)	7.5	7.0	6.3	5.9	5.3	6.6	6.4
NZ(-)	-5.0	-5.6	-5.6	-6.3	-3.1	-4.6	-7.3
NZ(+)	5.4	4.9	3.5	3.8	3.9	4.2	4.4
C20(-)	-6.2	-6.4	-4.8	-3.7	-3.3	-5.6	-4.4
C20(+)	3.8	3.3	3.1	3.3	5.2	3.5	3.5
TRP99:NE(-)	-5.8	-6.1	-5.8	-5.3	-5.0	-5.1	-5.0
TRP99(+)	4.9	5.5	4.9	4.8	4.4	4.5	4.4
TRP201(-)	-4.8	-5.0	-6.0	-5.4	-4.9	-5.2	-4.4
TRP201(+)	4.2	3.7	4.1	4.8	3.3	3.4	3.3
Wat2(-)	-5.6	-5.1	-5.2	-3.8	-3.0	-5.6	-4.4
Wat2(+)	3.7	4.4	3.6	3.2	2.7	3.9	3.8

The signs in the parentheses correspond to the signs of the difference features in the observed DMAPS. Cl1(+1),Cl1(+2),Cl1(+3) corresponds to the three positive peaks associated to the Cl^-1 next to the Schiff base. Cl1(+3) is the peak close to Thr102.

Table S3. Distances around the ion Cl^-1. The distances are measured in Angstrom.

distance	dark	1ps	2ps	50ps	100ps			
					0.17mJ/mm^2	0.90mJ/mm^2	2.63mJ/mm^2	6.49mJ/mm^2
Cl1-NZ:235	3.0	4.4	4.4	4.9	4.0	4.4	4.7	4.4
Cl1-Wat2	3.5	3.8	3.7	3.6	4.3	5.7	5.7	5.9
Cl1-ND2:98	3.3	4.3	3.7	3.2	4.3	4.2	4.0	3.2
Cl1-NE1:99	3.9	4.0	4.6	4.1	4.6	4.4	5.4	5.1
Cl1-OG1:102	3.4	4.4	4.2	4.1	3.2	3.1	3.1	2.8
NZ:235-OG:234	4.2	3.7	4.4	2.9	4.7	3.2	2.9	3.9

Cl1 or Cl^-1 is the Cl^- ion next to the Schiff base.
The atoms are specified in the format of Atom Name:Residue ID.

Movie S1.

The 3D movie of ClR from dark to 100 ps after flash illumination. The coordinated movement of the atoms is manifest by the retinal isomerization, as well as the movement of chloride ion, water, and protein residues. The Cl^- ion first becomes dissociated from the protonated Schiff base due to the retinal isomerization. From 50 ps to 100 ps, the Cl^- ion showed trend of moving towards the Thr102.
